# The Mapping Imaging Spectrometer for Europa (MISE)

**DOI:** 10.1007/s11214-024-01097-8

**Published:** 2024-10-09

**Authors:** Diana L. Blaney, Karl Hibbitts, Serina Diniega, Ashley Gerard Davies, Roger N. Clark, Robert O. Green, Matthew Hedman, Yves Langevin, Jonathan Lunine, Thomas B. McCord, Scott Murchie, Chris Paranicas, Frank Seelos, Jason M. Soderblom, Morgan L. Cable, Regina Eckert, David R. Thompson, Samantha K. Trumbo, Carl Bruce, Sarah R. Lundeen, Holly A. Bender, Mark C. Helmlinger, Lori B. Moore, Pantazis Mouroulis, Zachary Small, Hong Tang, Byron Van Gorp, Peter W. Sullivan, Shannon Zareh, Jose I. Rodriquez, Ian McKinley, Daniel V. Hahn, Matthew Bowers, Ramsey Hourani, Brian A. Bryce, Danielle Nuding, Zachery Bailey, Alessandro Rettura, Evan D. Zarate

**Affiliations:** 1 Jet Propulsion Laboratory, California Institute of Technology, Pasadena, CA USA; 2 Applied Physics Laboratory, Johns Hopkins University, Laurel, MD USA; 3 Planetary Science Institute, Tucson, AZ USA; 4 University of Idaho, Moscow, ID USA; 5 University of Paris, Orsey, Paris, France; 6 Cornell University, Ithaca, NY USA; 7 Bear Fight Institute, Winthrop, WA USA; 8 Massachusetts Institute of Technology, Cambridge, MA USA; 9 UC San Diego, San Diego, CA USA

**Keywords:** Europa, Europa Clipper, Composition, Spectrometer, Mapping

## Abstract

The Mapping Imaging Spectrometer for Europa (MISE) is an infrared compositional instrument that will fly on NASA’s Europa Clipper mission to the Jupiter system. MISE is designed to meet the Level-1 science requirements related to the mission’s composition science objective to “understand the habitability of Europa’s ocean through composition and chemistry” and to contribute to the geology science and ice shell and ocean objectives, thereby helping Europa Clipper achieve its mission goal to “explore Europa to investigate its habitability.” MISE has a mass of 65 kg and uses an energy per flyby of 75.2 W-h. MISE will detect illumination from 0.8 to 5 μm with 10 nm spectral resolution, a spatial sampling of 25 m per pixel at 100 km altitude, and 300 cross-track pixels, enabling discrimination among the two principal states of water ice on Europa, identification of the main non-ice components of interest: salts, acids, and organics, and detection of trace materials as well as some thermal signatures. Furthermore, the spatial resolution and global coverage that MISE will achieve will be complemented by the higher spectral resolution of some Earth-based assets. MISE, combined with observations collected by the rest of the Europa Clipper payload, will enable significant advances in our understanding of how the large-scale structure of Europa’s surface is shaped by geological processes and inform our understanding of the surface at microscale. This paper describes the planned MISE science investigations, instrument design, concept of operations, and data products.

## Introduction

### Purpose of This Paper

The first part of this paper describes the scientific context for the MISE investigation, along with its measurement approach to answer fundamental questions about Europa. The second part of this paper describes the MISE instrument design, calibration, and science operations. The intent for this paper is to provide information about the engineering aspects of MISE that will help in the analyses of data collected.

### MISE Capability Overview

MISE is a high-optical-throughput, push-broom imaging spectrometer that can provide effective observations on either a flyby or orbital mission and is designed to operate within Europa’s challenging radiation environment. The instrument provides a spectral range from 0.8 to 5 μm at 10 nm/channel, an instantaneous field of view (IFOV) of 250 μrad/pixel, and swath width of 300 active pixels. This corresponds to 25 m/pixel in a swath 7.5 km wide at 100 km altitude, and 10 km/pixel full disk images at a distance of 40,000 km. The 0.8 to 2.5-μm region is essential for quantifying hydrates and water ice, while the 3 to 5-μm region is required for detecting low abundances of organics, most radiolytic products, and discriminating salts from acid hydrates. These longer wavelengths can also be used to measure thermal emissions from geologically active regions, if present.

Because the instrument combines the techniques of mapping (imaging) and spectroscopy (compositional identification), it is highly proficient at linking geologic and exogenic processes to the composition and physical properties of materials found on the surface (Becker et al. 2024, this collection). MISE will be the second imaging spectrometer to observe Europa multiple times from relatively low altitude, the first being the Near Infrared Mapping Spectrometer (NIMS) on the Galileo spacecraft (Carlson et al. 1992). After the Galileo mission ended in September 2003, the study of Europa continued via distant observations with ground-based telescopes (e.g., Brown and Hand 2013; Ligier et al. 2016), the Hubble Space Telescope (HST) (e.g., Sparks et al. 2010; Becker et al. 2022) and, from 2022 on the James Webb Space Telescope (JWST) (e.g., Villanueva et al. 2023; Trumbo and Brown 2023). Ground-based telescope and HST measurements both confirmed and expanded upon Galileo NIMS discoveries about the surface composition, exploring chemicals such as sulfur species and hydrogen peroxide, which are detectable at UV-visible and infrared wavelengths respectively (e.g., Meyer et al. 1972; Loeffler and Baragiola 2005; Hand and Brown 2013; Trumbo et al. 2020; Becker et al. 2022). However, limitations to these post-Galileo observations of Europa are both spatial and temporal in nature; for example, high latitudes cannot be observed using Earth based assets, or from Earth–Sun Lagrangian locations such as by JWST. Additionally, the Jupiter-facing hemisphere is also very difficult to observe from those vantage points. In addition, compared with the capabilities of MISE, the spatial resolution of these distant observations is extremely low. MISE will be able to address those limitations over a broad spectral range, linking surface composition to geologic processes and gaining invaluable insights into the habitability of Europa’s subsurface ocean (Vance et al. 2023, this collection).

### MISE in Context of Other Instruments and Missions

Observations made by MISE will be complemented by observations from the other Europa Clipper investigations. Observations by the Europa Imaging System (EIS) (Turtle et al. 2024, this collection) will provide additional geologic context. The EIS Wide Angle Camera (WAC) provides regional context at pixel scales comparable to those of MISE and the EIS Narrow Angle Camera (NAC) provides detailed observations of individual landforms at pixel scales 25× higher than MISE. Both EIS datasets will include stereo observations. EIS will collect both monochromatic and color images; the color observations will be acquired in six broadband filters spanning ∼350 to 1050 nm, with the two longest-wavelength filters (IR1 and 1 μm) overlapping with MISE.

Europa Thermal Emission Imaging System (E-THEMIS) observations (Christensen et al. 2024, this collection) will complement MISE thermal measurements. E-THEMIS observations will be acquired at spatial scales comparable to MISE, in three spectral bands spanning 7 to 70 μm. Such observations will be sensitive to much colder temperatures than MISE. Detections of a thermal anomaly by both instruments would allow tight constraints on the temperature and area distribution, with MISE measuring radiance from the warmest areas and E-THEMIS measuring temperatures from cooler areas.

Observations by the MAss Spectrometer for Planetary EXploration/Europa (MASPEX) (Waite et al. 2024, this collection), the SUrface Dust Analyzer (SUDA) (Kempf et al. 2024, this collection), and the Europa Ultraviolet Spectrograph (Europa-UVS) (Retherford et al. 2024, this collection) will complement MISE compositional information. MASPEX and SUDA will provide in-situ measurements of the composition of volatiles and grains sputtered or ejected from the surface. SUDA detections will be traced back to the surface to provide a low-resolution surface composition probability map, whereas MASPEX observations will investigate the composition of Europa’s tenuous atmosphere. Europa-UVS observations will measure the composition of Europa’s atmosphere via ultraviolet (UV) emission and transmission and constrain the composition of its surface via UV reflectance. These observations will span 55 to 210 nm at ∼10× lower spatial resolution than the observations made by MISE.

The Moon and Jupiter Imaging Spectrometer (MAJIS) is an imaging spectrometer with a similar spectral range and resolution onboard the JUpiter ICy moons Explorer (JUICE) ESA mission. JUICE successfully launched on April 14, 2023, and so MAJIS is expected to operate in the Jupiter system in a similar time frame as MISE. The icy satellite measurements made by MAJIS will complement MISE science objectives, as MAJIS will primarily observe Ganymede during a nine-month orbital phase, and then make 21 flybys of Callisto. Furthermore, the two scheduled JUICE flybys of Europa will provide opportunities for cross-calibration between MISE and MAJIS.

## Science

### Science Goals

MISE has two science goals that feed into high-priority composition and geology-focused questions about Europa (described in more detail in Daubar et al. (2024, this collection) and Becker et al. (2024, this collection)). The first is to assess the habitability of Europa’s ocean by understanding the inventory and distribution of surface compounds . The second is to investigate the geologic history of Europa’s surface and search for areas that Vance et al. (2023, this collection) are currently active. In this section, we discuss the elements and compounds that are most relevant to Europa and how they can be detected and mapped by MISE (with key spectral signatures summarized in Table 1). There are also microphysical characteristics that can be assessed by MISE, such as the lattice configuration of water ice (i.e., crystallinity) as well as grain sizes of various materials. We also describe the capability that MISE has to enable compositional assessments over multiple spatial scales, which is important because a material can appear different when viewed in coarse spatial-scale datasets, such as disk-averaged observations, compared to finer scales. MISE will collect observations of Europa’s surface composition over three orders of magnitude of scale, from 10s of kilometers per pixel to 10s of meters per pixel.

**Table 1:** Key Absorption Features Detectable by MISE

Compound	Wavelength position(s) and associated spectral properties	References	Comments
H_2_O_2_	2.76 μm (OH str), 3.5 μm^*^	Carlson et al. 1999; Loeffler and Baragiola 2005	3.5-μm band is seen on Europa and characterized in the lab.
CO_2_	2.7 μm^*^, 4.26 μm^*^ (4.25- & 4.27-μm double-peaked structure)	Villanueva et al. 2023; Trumbo and Brown 2023	From JWST data (2.7-μm v1+v3 band is very narrow; 4.26-μm v3 band has two minima)
^13^CO_2_	4.38 μm^*^	Villanueva et al. 2023	v3 asymmetric stretch; from JWST data and is quite narrow
SO_2_	4.07 μm, 4.37 μm	Nash and Betts 1995	
H_2_O (crystalline and amorphous)	1.5 μm^*^, 1.65 μm^*^, 2 μm^*^, 3 μm^*^, 3.1 μm (Fresnel)^*^, 4.5 μm^*^	Carlson et al. 2009 (Europa book chapter)	Various water bands. 1.65-μm absorption indicates crystalline ice. There is also a Fresnel reflection peak at 3.1 μm
Unidentified	2.07 μm^*^	Brown and Hand 2013; Davis et al. 2023	Epsomite originally suggested (Brown and Hand 2013); also can be matched via mixtures of various Cl-bearing salts (Ligier et al. 2016); spatial distribution suggests unrelated to salts (Davis et al. 2023); remains unidentified and only seen on trailing side
Unidentified	3.78 μm^*^	Trumbo et al. 2017	Only on trailing side
Carbonates	3.4-3.5 μm, 3.9-4 μm	De Angelis et al. 2019; Huang and Kerr 1960; Harner and Gilmore 2015	Seen in some primitive asteroid samples (e.g. Kaplan et al. 2020)
Sulfates	1.5^*^, 2^*^, 3 μm asymmetric absorptions (hydrated phases) 3.8-5 μm	Clark et al. 2007, Cloutis et al. 2006; Bishop et al. 2014; De Angelis et al. 2021	Asymmetric water bands for hydrated phases Sulfate overtone/combination bands at longer wavelength
Acid Hydrates	1.5^*^, 2^*^, 3 μm asymmetric absorptions	Carlson et al. 2009	Asymmetric water bands
Ureas	2.91-3.13 μm	Socrates 2001	NH stretch
Alkanes (aliphatic)	3.36-3.52 μm	Socrates 2001	OCH_3_ and NCH_3_ at long end (3.45-3.55 μm)
Alkanes (aromatic)	3.22-3.33 μm	Socrates 2001	CH stretch (up to 5 peaks, decrease w/increase in substitution)
Alkenes	3.17-3.33	Socrates 2001	CH stretch
Alkynes	2.99-3.05 μm	Socrates 2001	CH stretch
Conjugated alkynes (w/COOH or COOR)	4.43 μm	Socrates 2001	
Primary amines (aliphatic)	2.90-3.16 μm, 3.40-3.43 μm	Socrates 2001	NH_2_ stretch, CH_2_ stretch
Primary amines (aromatic)	2.92-2.99 μm	Socrates 2001	Asymmetric NH_2_ stretch
Secondary amines (aliphatic)	3.42-3.59 μm	Socrates 2001	Symmetric CH_3_ stretch
Secondary amines (aromatic)	2.90-2.94 μm, 3.55 μm	Socrates 2001	C-H stretch of N-C-H at 3.55 μm more intense than alkyl
Tertiary dimethyl amines (−N(CH_3_)_2_)	3.31-3.42 μm, 3.42-3.61 μm	Socrates 2001	Asymmetric CH_3_ stretch, Symmetrical stretch CH3 stretch
Imines	2.94-3.23 μm	Socrates 2001	NH stretch
Primary amides	2.83-2.88 μm	Socrates 2001	Asymmetric N-H stretch
Secondary amides	2.89-2.93 μm	Socrates 2001	Asymmetric N-H stretch
Imides (solid phase)	3.05-3.13 μm	Socrates 2001	Asymmetric N-H stretch
Diazoketones (−CO-CN_2_-)	4.76-4.87 μm	Socrates 2001	
Nitriles	4.43-4.55 μm	Socrates 2001	Acetylene (C≡C) also absorbs here
Isocyanates (−N=C=O)	4.35-4.44 μm	Socrates 2001	Asymmetrical NCO stretch; aryl isocyanates 4.38-4.42
Isothiocyanates (−N=C=S)	4.65-5.03 μm	Socrates 2001	Broad asymmetrical stretch, usually doublet
Hydroxyl (intermolecular)	2.82-3.10 μm	Socrates 2001	Usually broad but may be sharp; frequency is concentration-dependent
Primary alcohols	3.34-3.52 μm	Socrates 2001	Asymmetric CH_2_ stretch
Phenols	3.08-3.33 μm	Socrates 2001	
Phenols (substituted)	2.73-3.09 μm	Socrates 2001	
Ethers (aliphatic, −OCH_3_)	3.34-3.38 μm, 3.45-3.52 μm	Socrates 2001	Asymmetric −CH3 stretch, Symmetric −CH_3_ stretch
Methyl aromatic ethers (=C-O-CH_3_)	3.53-3.55 μm	Socrates 2001	CH_3_ stretch
−OH (associated carboxylic acids)	3.00-4.00 μm	Socrates 2001	OH stretch w/H-bonding
Peroxy acids (CO-OOH)	3.05 μm	Socrates 2001	Intramolecular OH stretch
Methyl esters (saturated)	3.28-3.36 μm, 3.30-3.39 μm	Socrates 2001	Asymmetric CH_3_ stretch
Methyl esters (unsaturated)	3.27-3.21 μm, 3.36-3.42 μm	Socrates 2001	Asymmetric CH_3_ stretch
Acetates	3.34 μm	Socrates 2001	Asymmetric CH_3_ stretch
Pyridines, pyrimidines	3.21-3.32 μm	Socrates 2001	CH stretch; pyridinium salts shifted to 2.99-3.12 μm
Melamines	2.86-3.23 μm	Socrates 2001	NH_2_ stretch (variable intensity)
Pyrroles	2.94-3.33 μm	Socrates 2001	Broad NH stretch, H-bonded
Furan derivatives	3.14-3.33 μm	Socrates 2001	=C-H stretch
Thiophenes	3.21-3.33 μm	Socrates 2001	=C-H stretch
Chloroalkanes (−CH_2_Cl)	3.29-3.50 μm	Socrates 2001	Asymmetric CH_2_ stretch
Dichloroalkanes (−CHCl_2_)	3.31-3.36 μm	Socrates 2001	CH stretch
Primary sulphonamides (−SO_2_NH_2_)	2.95-3.08 μm	Socrates 2001	Asymmetric + symmetric NH stretch
−CH_2_S−	3.35-3.43 μm	Socrates 2001	Asymmetric CH_2_ stretch
−CH_2_SH	3.35-3.41 μm, 3.40-3.50 μm	Socrates 2001	Asymmetric CH_2_ stretch, Symmetric CH_2_ stretch
−CS-NH-CH_3_	3.01-3.14 μm, 3.33-3.42 μm, 3.42-3.55 μm	Socrates 2001	NH stretch, CH_3_ asymmetric stretch, CH_3_ symmetric stretch
OCS	4.89 μm	Ferrante et al. 2008	v3 band of OCS in H_2_O or CO_2_ ices. 4.95 μm in pure OCS
OD stretch in ice	4.12-4.13 μm	Clark et al. 2019	
OD stretch in other compounds, not ice	3.5-4.1 μm	Clark et al. 2019	

^*^Indicates that the band has been detected on Europa

MISE is designed to enable key discoveries about Europa, including understanding of how water ice is distributed and the composition of the non-ice materials, such as frozen salts, acids, and organics, with the ultimate goal of linking surface composition to subsurface ocean habitability. Observations by MISE will also be used to determine isotopic ratios for some surface materials, including the D/H ratio of water ice. The isotopic ratios can help constrain origin and evolution of the material. Additionally, while MISE does not operate throughout the traditional thermal infrared wavelengths, it will acquire observations useful for detecting thermal signatures on the surface of Europa. Finally, the high spatial resolution observations by MISE over all latitudes of Europa from within the Jovian system will complement the coarser spatial resolution data acquired by terrestrial and other space-based assets.

### Understanding the Inventory and Distribution of Surface Compounds

#### Overview

On a global scale, the surface of Europa is dominated by water ice and at least one highly hydrated material. The leading hemisphere contains a higher concentration of water ice than the trailing hemisphere and the high latitudes are also primarily water ice (Calvin et al. 1995). The equatorial surface, particularly the trailing hemisphere, contains significant amounts of some unknown, non-ice materials (e.g., Clark and McCord 1980). McEwen (1986) also remarked on the differences between the leading and trailing hemispheres, including that the leading hemisphere is much brighter and icier. The composition of the non-ice hydrated material on Europa was more rigorously identified as either (or a mixture of) hydrated salts, interpreted primarily as sulfates (McCord et al. 1999), or hydrated sulfuric acid (Carlson et al. 1999). Salts would imply an endogenic origin and would reveal information about Europa’s subsurface ocean. Sulfuric acid hydrate would imply exogenous effects such as the bombardment of the surface by charged particles, including sulfur ions, whose source can be traced mainly to the volcanic moon Io (as summarized in Paranicas et al. 2009). More recent ground-based observations appeal to both processes, with compelling evidence for a different salt (sodium chloride; NaCl) on the leading hemisphere that is irradiated by charged particles (Trumbo et al. 2019a, 2022), making it spectrally active in the visible (e.g., Hand and Carlson 2015). Other Earth-based observations and reanalysis of Galileo NIMS data suggest sulfuric acid hydrate as the dominant non-ice material on the trailing hemisphere, though contributions from hydrated salts remain possible (Dalton et al. 2012; Brown and Hand 2013; Fischer et al. 2015; Ligier et al. 2016; Mishra et al. 2021). The 1–2.5 μm and 3–4 μm spectral information that MISE will obtain will surpass the spectral resolution of the NIMS data sets (a full width at half maximum (FWHM) of 26 nm) and the spatial resolution of Earth-based observations, enabling determination of both the physical and chemical composition of the ice and/or acid hydrate to infer origin and possibly provide insights into the habitability of the underlying ocean. Table 1 lists observable spectral features of specific compounds that MISE may detect to provide these constraints.

Another aspect of Europa’s surface is the alteration by co-rotating plasma and high energy charged particles, sometimes associated with >100 keV ions and electrons. These agents catalyze new physical and chemical pathways in surface materials that include liberation of water molecules and the creation of new products, such as hydrogen peroxide and polymeric sulfur, via radiolysis. At higher energies, irradiation is linked to a transition in water ice from the crystalline to amorphous state, and to other physical changes such as the compaction of grains (Palumbo 2005; Raut et al. 2007; Famá et al. 2010). MISE data analysis will be shaped by the nature of the geology as well as the precipitation patterns of plasma and particles onto regions with varying levels of water ice in the uppermost layer. Since precipitation patterns of plasma and particles are fairly well understood, MISE will be able to observe the consequences of radiolytic modification of ice and non-ice components by comparing composition to the predicted level of exposure to magnetospheric particles.

#### Water Ice

Much of Europa is covered in ice, with the highest concentrations of water ice on the leading hemisphere and at high latitudes (e.g., Grundy et al. 2007; Carlson et al. 2009; Brown and Hand 2013; Ligier et al. 2016). Although water ice is endogenic, its concentrations and physical characteristics are shaped by several factors. For example, the leading hemisphere is brighter and icier than the trailing, in part because of preferential bombardment of the leading hemisphere by bolides that excavate subsurface ice, as well as preferential sputtering of water ice from the trailing hemisphere (McEwen 1986; Paranicas et al. 2009; Plainaki et al. 2012; Cassidy et al. 2013). The trailing hemisphere also receives a higher flux of Jovian charged particles than the leading hemisphere, including the majority of Iogenic sulfur ions, which makes the ice less pure via direct contamination, increased charged-particle sputtering, and sulfur radiolysis reactions (e.g., McEwen 1986; Carlson et al. 2002; Plainaki et al. 2012; Cassidy et al. 2013). Observations collected by MISE will be used to create a global map of the crystallinity of the ice via the shape of the 2-μm band (Mastrapa and Brown 2006) and the compositions of the non-ice materials on Europa’s surface, within the context of Europan geology. This will improve our understanding of how the current composition on Europa’s surface came to be established.

MISE can identify and map the crystalline and amorphous states of water ice more accurately than NIMS did and do so at higher spatial resolution and with greater global coverage than Earth-based observations (Fig. 2). Almost all water ice in the Solar System is in the crystalline state, but much of the ice in the very top layer of Europa’s surface is not. Being exposed to particle bombardment from the Jovian system environment, the crystal structure is in an apparent amorphous state, at least for ice on the outer surface of grains (Hansen and McCord 2004). Ice below the penetration depth of most UV photons and ions (i.e., <10 μm depth) retains the spectral structure associated with crystalline ice (Hansen and McCord 2004; Ligier et al. 2016). MISE will be able to more precisely determine how the two principal states of ice are spatially distributed on the surface and, by working with other instruments such as E-THEMIS (Christensen et al. 2024, this collection), determine how factors such as thermal drivers influence the balance between amorphous and crystalline ice. Radiation patterns on Europa have been coarsely predicted (e.g., Paranicas et al. 2001) and will be improved by simulations so that MISE maps of amorphous ice can be compared with models of precipitation of energetic charged particles to understand formation mechanisms. Since many pathways exist for ice to transition from crystalline to amorphous, constraints on impacts and other large-scale features that are derived from the observations collected by MISE will be critical.

#### Non-ice Constituents

Acid hydrate and hydrated salts are the leading contenders for the composition of the bulk of the non-ice, but hydrated, material on Europa (e.g., McCord et al. 1999; Carlson et al. 1999). The hydrate of sulfuric acid (H_2_SO$_{4}\boldsymbol{\cdot}$nH_2_O) is the most likely candidate for the acid hydrate component (Carlson et al. 1999, 2005; Brown and Hand 2013). Its origin has been hypothesized as resulting from subsequent evolution of the implanted Iogenic sulfur (Carlson et al. 1999, 2002, 2005). Hydrated salts would imply a completely endogenous source. Broadly speaking, acid hydrates on Europa are believed to result from the implantation and radiolysis of exogenous sulfur (e.g., Carlson et al. 1999), whereas hydrated salts are believed to be endogenic (e.g., McCord et al. 1999). Grundy et al. (2007) found that the trailing side of Europa, which faces the plasma ram, consists primarily of non-ice material, supporting the hypothesis that external agents play a role either by implanting Iogenic materials and/or by preferentially removing water ice through their action. Trumbo et al. (2019a) have shown that NaCl exists on the leading hemisphere of Europa, potentially derived from interior-surface exchange during the formation of large-scale regions of chaos terrain and spatially separated from the heavy bombardment of the trailing hemisphere by Iogenic sulfur.

Because Europa is deep within the radiation belts of Jupiter, it is important to recognize that molecular signatures at various wavelengths may be altered by the environment. Hand and Carlson (2015) for example have pointed out that the color of some salts under the action of tens of keV electrons would be different than samples with no exposure to such agents. They also note that some targets that are invisible to many wavelengths can have irradiation products that are accessible to those wavelengths.

##### Hydrated Minerals and Acid Hydrates

Hydrated materials, whether endogenous or of exogenous origin, can be thermally stable at Europa’s surface temperature for hundreds of millions of years and potentially for geologic time (McCord et al. 2001). Sodium sulfates and carbonates are less thermally and radiolytically stable and may be preferentially removed (especially from the trailing hemisphere) compared to magnesium-bearing sulfates, with the caveat that the study investigated only thermal desorption and electron stimulated desorption, and not sputtering by ion bombardment.

These hydrated minerals and their brines can be distinguished from water ice signatures via characteristic distortions of the water bands (e.g., McCord et al. 1998; Carlson et al. 1999; Orlando et al. 2005) and possibly via narrower absorptions not present in water ice (e.g., Hanley et al. 2014). Although interpretations of ground-based spectra and re-analyses of NIMS spectra across the 1.5–2.5 μm range have favored hydrated sulfuric acid as the dominant non-ice component of the trailing hemisphere (Dalton et al. 2012; Fischer et al. 2015; Ligier et al. 2016; Mishra et al. 2021), endogenic hydrated salts associated with geologic terrains remain a possible component. Ground-based spectra from the W.M. Keck Observatory show a previously undetected distinct signature, potentially of hydrated magnesium sulfate (epsomite, MgSO$_{4}\cdot $7H_2_O) at 2.07 μm on the trailing hemisphere of Europa (Brown and Hand 2013). As the feature is spatially correlated with the presence of radiation products like sulfuric acid and SO_2_, it is possible that the sulfate resulted from the radiation induced oxidation of MgCl_2_ in the presence of water ice and/or other hydrated materials (Brown and Hand 2013). However, more recent analysis of the same absorption feature in spectra obtained by the Very Large Telescope (VLT) reveal no correlation with endogenic geologic terrains, instead suggesting an unknown exogenic species involving sulfur radiolysis, but not endogenic salts (Davis et al. 2023). Thus, the identity of the 2.07 μm band remains undetermined.

The acquisition of high signal-to-noise (SNR) spectra over the 0.8 to 5 μm region will enable MISE to discriminate between various compositions of salts by detailed inspection of the 1–2.5 μm region and the discrimination between sulfates and acid hydrates by inspection of the 3 to 5-μm region, as water-ice has a reflection peak near 3.6 μm (Mastrapa et al. 2009) and hydrated salts peak near 3.8 μm (e.g., bloedite and epsomite; Clark et al. 2007), but acid hydrate has no reflection peak in this spectral region (e.g., Carlson et al. 2009). On Europa, the mid-IR reflection peak varies from ∼3.6 μm at the icy high latitudes to ∼3.7 μm at low latitudes on the leading and trailing hemispheres, but it is more subdued on the trailing hemisphere (Fischer et al. 2017; Trumbo et al. 2017).

##### Chlorides

As shown in studies of Earth’s sea water, the exact salt composition of ocean-derived surface material is diagnostic of different pathways for the geochemical evolution of the ocean waters (e.g., Zolotov et al. 2009), though the surface salt assemblages will also be influenced by varied precipitation during freezing (e.g., Zolotov and Shock 2001; Vu et al. 2016; Johnson et al. 2019) and other ice-shell processes. Europa is believed to have multiple pathways by which the subsurface communicates with the surface, each of which may yield a different distribution of salts at the surface, making the investigation of salts crucial for achieving the Europa Clipper science goals. Though initial interpretations of endogenic salts on Europa from 1.5–2.5 μm NIMS spectra focused primarily on hydrated sulfates (e.g., McCord et al. 1998, 1999), analyses of ground-based data at these wavelengths (Brown and Hand 2013; Fischer et al. 2015, 2017) and of UV-visible spectra (Trumbo et al. 2019a, 2022) favor chlorides. Ligier et al. (2016) also found that magnesium-bearing chlorinated salts fit their near-infrared (NIR) data better than sulfates, although their fits are not unique. Chlorides, especially halides, are not spectrally active in the NIR aside from water-related absorptions that are spectrally similar to water ice, but form color centers—optically active radiation-induced crystal defects—under particle irradiation (e.g., Hand and Carlson 2015; Hibbitts et al. 2019; Brown et al. 2022). Indeed, absorption features due to color centers in NaCl have been detected on the leading hemisphere with HST (Trumbo et al. 2019a, 2022). Hand and Carlson (2015) point out that color centers in such NaCl could provide a metric for how long this salt or others have been on the surface in different regions, although photobleaching by absorbed sunlight may complicate this by limiting the intensity and complexity of the color centers (Denman et al. 2022). However, some optically active color centers of irradiated halides and other salts can extend into the NIR (Hand and Carlson 2015; Hibbitts et al. 2019). Observations by MISE will enable detection of the NIR signatures of these color centers, if present, and the generation of spatial maps that will complement color maps from EIS (Turtle et al. 2024, this collection) and help identify areas that include chloride salts.

##### Organics

Understanding the inventory of organics on Europa is critical for assessing its habitability (Vance et al. 2023, this collection). An absorption feature consistent with the CH_2_/CH_3_ stretch of aliphatic organics is found on Ganymede and Callisto (e.g., McCord et al. 1998) and absorptions interpreted as both aliphatic and aromatic hydrocarbons are found in spectra of other icy bodies (e.g., Cruikshank et al. 2014). Organics may also be present on Europa’s surface, originating from impact processes, photochemistry of implanted phases, and/or Europa’s ocean. Observations by MISE will be used to generate maps of the distribution and composition of organics that will help distinguish between materials of exogenic or ocean provenance. For example, organics that originate in Europa’s ocean should be spatially associated with features and terrains that are suspected to be formed through movement of material up through Europa’s shell, including bands, ridges, and chaos. Ocean-derived organics may also be associated with nonorganic phases, such as hydrated salts and carbon dioxide.

In addition to the identification of organics, the ability to distinguish between various types of organic compounds is desired. The relative abundances of hydrocarbons (i.e., CH-bearing species) with distinct spectroscopic signatures (Table 1; Clark et al. 2009, 2010, 2014; Cruikshank et al. 2014, 2020; Kokaly et al. 2017) can be compared against both the cometary abundance pattern and that predicted for simple abiotic processes, such as Fischer-Tropsch reactions (C.P. McKay 2004; Summons et al. 2008). If the dominant composition of organics that are determined (through spatial and other analyses such as correlation with salts and thermal anomalies) to have been extruded onto the surface from a subsurface reservoir differs from the composition expected of abiotic systems, this could be evidence that the ocean is actually inhabited (Hand et al. 2009; Zolotov et al. 2009). However, this is a weak test for life because it may be possible for abiotic organics to have been altered by radiation in a way to mimic a deviation from an abiotic pattern.

Once exposed on Europa’s surface, radiation could cause organics to degrade, depending on the strength of the relevant bonds, breaking down and removing structural bonds diagnostic of the initial organic composition. Experiments involving the irradiation of short-chain hydrocarbons in water ice have also seen the production of refractory long-chain aliphatic hydrocarbons (Hand and Carlson 2012) and additional laboratory work may further inform the timescale and degree of expected degradation. Compounds could be created due to radiation, as well as destroyed. Much more lab work is also needed to better inform the range of materials that could be present on Europa’s surface. Less irradiated surfaces (such as fresher surfaces or those more shielded from radiation) could contain organic molecules that retain more of their spectral structures (Paranicas et al. 2009; Nordheim et al. 2018). These areas can be at the scale of individual geologic features such as plume vents or portions of ridges.

#### Trace Materials

##### Hydrogen Peroxide

Galileo NIMS observed trace amounts of hydrogen peroxide (H_2_O_2_) on Europa’s leading hemisphere (Carlson et al. 1999) and Hand and Brown (2013), using disk-integrated Keck observations that are spanned in wavelength by MISE, confirmed this observation. The latter reported that concentrations of H_2_O_2_ on Europa’s surface were lowest on the trailing hemisphere. It is believed that H_2_O_2_ is a radiolysis product of ice that can be formed by a number of agents (see summary in Hand and Carlson 2011). Since the trailing hemisphere of Europa is likely to be the most heavily irradiated, this raises the question of why the peroxide concentrations are lowest there. Two possible explanations are that the amount of water ice on the trailing hemisphere is lower than many other locations on the surface (Grundy et al. 2007; Ligier et al. 2016) and/or that H_2_O_2_ reacts with SO_2_, also formed on the trailing hemisphere, to form SO_4_ and the related acid hydrate (e.g., Loeffler and Hudson 2013).

While H_2_O_2_ is very unstable (e.g., Hudson and Moore 2006) and only observed in trace amounts (e.g., Carlson et al. 1999), it is still a valuable marker of a region of the surface that is being bombarded by plasma and particles. Trumbo et al. (2019b) have partially mapped it across the leading hemisphere using Keck adaptive optics (AO) observations, finding the largest band strengths specifically at low latitudes and thus seemingly anti-correlated with water ice abundance. Corotating plasma does not interact as much with low latitudes on Europa’s leading hemisphere, but these regions are preferentially bombarded by ≥20 MeV electrons that move against the co-rotation direction (Nordheim et al. 2018), and UV photons may also contribute. The multi-year Keck dataset of Trumbo et al. (2019b) also exhibits some evidence for temporal variations in H_2_O_2_ concentration. Peroxide formed by UV is expected to have a more predictable concentration pattern that would be correlated with the locus of subsolar points, but it is not clear that solar insolation can explain the temporal changes. Observations by MISE will be used to detect H_2_O_2_ and create distribution maps of its 3.5-μm absorption, including over time, using repeated global scale observations from successive flybys. Doing so will establish which particles (e.g., photons, plasma, radiation, etc.) are most dominant in the production of peroxide on the surface and confirm how surface composition can enhance or retard H_2_O_2_ growth. Peroxide may also be relevant to the organic chemistry of Europa. For instance, it is an oxidant (i.e., can receive an electron) and it could therefore be potentially involved in important chemical pathways, such as being destructive to some compounds or indirectly revealing the presence of others. Comparing H_2_O_2_ distribution maps against predicted patterns of weathering by various agents can therefore be used to test which processes are dominant on Europa.

##### Ozone

Laboratory work on the formation of ozone on the icy satellites through irradiation of thin films (e.g., Bennett and Kaiser 2005) has confirmed that the presence of O_2_ in water ice can be enabling to ozone production (Cooper et al. 2008). As yet, however, no O_3_ has been detected on Europa, and Noll et al. (1996) have shown ozone to be more prevalent on Ganymede. This difference may be the result of Europa’s surface not being shielded by a permanent magnetic field and the flux of Iogenic sulfur being higher since the moon is much closer to Io. Both increase the likelihood of SO_2_ and H_2_S reactions with ozone, which are thermal pathways toward depleting ozone (Loeffler and Hudson 2016; Tribbett and Loeffler 2022). Additionally, chemical processes on Ganymede that form ozone may have longer to develop since Ganymede’s low latitude regions are less heavily weathered by particles in the Jovian magnetosphere.

##### Molecular Oxygen

Molecular oxygen is an additional marker for water alteration processes and is more likely to be retained than molecular hydrogen, which is thought to escape very readily from the surface and atmosphere. One pathway for the alteration of water ice under different bombardment conditions is the formation of H_2_O_2_, as discussed above. Peroxide is rapidly broken down into molecular hydrogen and oxygen in the ice. Another pathway by which molecular oxygen forms is through the water group pickup (ionization followed by acceleration) initiated by magnetospheric electrons or sunlight. Modeling of these processes (e.g., Smyth and Marconi 2006) has shown that atomic and molecular oxygen is more tightly bound to Europa, while hydrogen spreads out. Hall et al. (1998) found high column densities of molecular oxygen at Europa, and Spencer and Calvin (2002) detected an absorption feature at visible wavelengths (577 nm) diagnostic of trapped O_2_ in the surface.

##### Carbon Dioxide

In the Jovian system, carbon dioxide (CO_2_) was detected by NIMS on Europa, Ganymede, and Callisto (e.g., McCord et al. 1998; Hibbitts et al. 2000, 2003) via its strong asymmetric vibration ($\nu _{3}$) band near 4.26 μm (McCord et al. 1998; Carlson et al. 2009). However, as high SNR NIMS observations beyond 2.5 μm were extremely limited, little about this CO_2_, including its origin, physical state, and exact band position, was constrained. Observations made by JWST have confirmed the presence of solid-state CO_2_ on Europa, detecting two absorption minima centered near 4.25 and 4.27 μm within the $\nu _{3}$, consistent with complexed CO_2_ and the position expected of crystalline CO_2_ ice, respectively, along with detecting a weaker CO_2_ feature near 2.7 μm (Trumbo and Brown 2023; Villanueva et al. 2023). The host material for the complexed CO_2_ has not been identified, but trapping within water ice, hydrated salts, or other non-ice materials warrant investigation. Additionally, crystalline CO_2_, which has a high vapor pressure at Europa’s temperatures in the range ∼80–130 K, is not expected to be stable at Europa’s surface conditions, except at the poles (Carlson et al. 2009). Thus, the exact mechanisms behind the observed CO_2_ bands are still uncertain. However, these CO_2_ bands are strongest in Tara Regio, a large-scale, low-latitude region of disrupted chaos terrain and the region containing the strongest NaCl signatures, suggesting a possible ocean origin for the CO_2_ or radiolytic formation of CO_2_ from recently exposed carbon-bearing species (Trumbo and Brown 2023; Villanueva et al. 2023). With the high SNR in the 4-μm region, global coverage, and high spatial resolution, observations by MISE will enable investigation of the origin of CO_2_ as either endogenic, such as outgassing of CO_2_-clathritic water ice that is hypothesized to account for the CO_2_ on Callisto and Ganymede (Hibbitts et al. 2000, 2003), or as a result of a plume deposit, such as occurs on Enceladus (Brown et al. 2006; Combe et al. 2019).

##### Isotopic Ratios

Isotopic ratios can help constrain origin and evolution of a compound on a planetary surface. The inner Solar System, including Earth, Mars, Vesta, C- and S-type asteroids has a narrow range of D/H ratios, about 6 times higher than proto-solar, 1.5 × 10^−4^. The D/H of the bulk Earth is 1.49 (± 0.03) × 10^−4^ (Lecuyer et al. 1998). The current interstellar medium (ISM) is on the order of 0.016 × 10^−4^, lower today due to the destruction of deuterium by stars since the Big Bang (Robert 2006). Models of solar system formation have D/H increasing with distance from the Sun (see summary in Clark et al. 2019). However, Clark et al. (2019), using reflectance spectroscopy to determine D/H ratios in ice, found the D/H in Saturn’s rings and satellites was near terrestrial and significantly lower than model predictions. Clark et al. also showed that the carbon 12 to 13 ratio in CO_2_ (trapped or solid) can also be derived with reflectance spectroscopy at Cassini Visible-Infrared Mapping Spectrometer (VIMS) resolution (FWHM ∼16 nm). Using JWST spectra, Villanueva et al. (2023) measured the ^12^CO_2_/^13^CO_2_ ratio to be consistent with the Earth inorganic standard and with measurements for Iapetus and the asteroid Ryugu. MISE has a higher spectral resolution than VIMS and can be used to measure these ratios, and potentially others, including oxygen isotopes for materials with sharp absorption bands (the absorptions in water ice are too broad to determine oxygen isotopic ratios). By measuring reflectance spectra of fresh deposits of water from the ocean, if they exist, the isotopic ratios of ocean water might be determined. These measurements will help constrain the origin and evolution of water in and on Europa. With the complexities of mixtures with salts, acids, and other compounds, deriving isotopic ratios will need radiative transfer models to unscramble the nonlinear mixture effects on reflectance spectra. Such analyses could be achieved on some local spots, but laboratory work would be needed to derive methods that could be applied to imaging spectroscopy data to provide maps of isotopic ratios.

#### Jovian Magnetospheric Particle Bombardment Effects on Surface Composition

Charged particles trapped in Jupiter’s magnetosphere can modify constituents in Europa’s atmosphere and surface, as discussed above. Figure 3 shows how various magnetospheric agents may reach Europa’s surface. Less energetic particles may be stopped in the patchy atmosphere but more energetic ions and electrons over a wide energy range will reach the surface. For example, 100 eV electrons can ionize the target atoms and molecules expected in Europa’s atmosphere (Johnson 1990). A critical process for protons is charge-exchange, which would render the primary a neutral. This would be an important loss process for protons below 100 keV. High energy particles will pass easily through the atmosphere with some energy loss or scattering. A ballpark estimate is that all >10 keV electrons and >100 keV protons could reach the surface, along with many particles below those energies.

Charged particles that reach the surface can modify Europa’s surface composition via radiolysis, whereby irradiated species are chemically modified, forming new components. For example, the formation of H_2_O_2_ from H_2_O is a classic radiolytic process. Charged particle bombardment will also sputter surface molecules off Europa’s surface, which then populate its atmosphere. Another example is that MeV-energy sulfur can lead to the ejection of a water molecule by interacting with the electrons in the target or by transferring energy to target nuclei. Johnson and Sundqvist (2017) have suggested that even organics can be liberated intact by sputtering processes.

Surface constituents whose origins may be linked to external sources have been described by Carlson et al. (2009). For example, if hydrated sulfuric acid is indeed present on the surface of Europa, it is likely to be exogenic. While plasma and charged particles impact all of Europa’s surface, there is likely more precipitation onto the trailing hemisphere than the leading side. Carlson et al. (2009) describe several other compounds containing sulfur that could be present on Europa, including SO_2_ and certain sulfur allotropes, the UV-visible signatures of which have recently been mapped across the trailing hemisphere with spectra from HST (Trumbo et al. 2020; Becker et al. 2022). Another absorption band near 3.78 μm on the trailing hemisphere may imply an additional unidentified radiolysis product, thereby adding to that inventory (Trumbo et al. 2017). Observations by MISE will make more precise measurements of the sulfur inventory on Europa’s surface and map this and other markers more fully.

The way the magnetosphere of Jupiter interacts with the Europa environment is complicated because of the electromagnetic fields that are present near the body (e.g., the Alfvén wing current system). The high-level picture of the interaction is as follows. Cold plasma will overtake Europa in its orbit and preferentially bombard the trailing hemisphere. Energetic ions will likely reach the whole surface. Electrons are predicted to form a lens shape on the trailing hemisphere if their energies are between about the hundreds of keV and ∼20 MeV. The electrons just above 20 MeV should form a lens shape on the leading hemisphere (see Fig. 4). The electromagnetics of Europa, which can be described for example by magnetohydrodynamic (MHD) simulation, will alter this simple picture and may show more complicated energy-dependent patterns of electrons.

The precipitation of Iogenic oxygen or sulfur does not alter underlying geology of the trailing hemisphere but may obscure or modify the associated endogenic materials. Indeed, ground-based observations have noted that regions of large-scale geology on the more pristine leading hemisphere appear spectrally and compositionally different from their trailing-hemisphere counterparts (Fischer et al. 2015, 2017; Trumbo et al. 2019a, 2020, 2022). The global coverage at much higher spatial resolution provided by MISE will enable detailed comparisons between terrain types on both hemispheres and, thus, enable a more robust deconvolution of endogenic and exogenic effects on the surface composition than has been achieved using ground-based datasets and NIMS.

#### Meteoritic Materials

Another agent that weathers Europa is dust from comets and other sources. Dust grains in a magnetosphere can become charged and, if they are very small (less than about 0.1 μm in radius), flow over Europa like the plasma (first reaching the trailing hemisphere). By contrast, larger grains evolve more by gravitational forces than electromagnetic ones (Grün et al. 1984; Burns et al. 2001; Horányi and Juhász 2010; Jontof-Hutter and Hamilton 2012; Liu et al. 2016). The bombardment patterns onto Europa’s surface therefore depend on both the size of the grains and the source of the grains. Potential dust sources include Io, Ganymede, Callisto, Jupiter’s irregular satellites, and even objects outside the Jovian system (Bottke et al. 2013; Liu and Schmidt 2019). Since exogenic dust grains from any of these sources could deliver organics onto Europa’s surface, discrimination of whether organics were carried onto Europa by dust or are endogenic requires identifying the source of the dust and the likely bombardment pattern. MISE will be a critical asset in these investigations.

### Investigating the Geologic History of Europa’s Surface and the Search for Current Activity

In conjunction with EIS and other instruments on Europa Clipper, MISE will investigate the compositional and geologic processes that have affected Europa’s surface and those that continue to do so, including areas that may be currently active. Observations by MISE will be used to test various hypotheses (Collins et al. 2009; Daubar et al. 2024, this collection) that have been proposed to explain its surface features. For example, the formation of Europa’s chaos terrain has been hypothesized to be due to: *melt through* (Carr et al. 1998; Greenberg et al. 1999), *diapirism* (Pappalardo et al. 1998, 1999, 2004), *brine mobilization* (Head and Pappalardo 1999), *injection of sills* (Crawford and Stevenson 1988; Michaut and Manga 2014), *impact* (Billings and Kattenhorn 2003; Cox et al. 2005), or *shallow subsurface water lenses* (Schmidt et al. 2011). Analysis of data collected by MISE will be used to test these formation hypotheses (Daubar et al. 2024, this collection) by comparing the chemistry exposed in the matrix of the chaos terrains and of the lenticulae (Fig. 5) within and between individual landforms. For instance, direct ocean interactions such as melt-through or impact could have surface compositions more closely reflecting the ocean’s composition. Brine mobilization and diapirism models suggest the dominance of fractional crystallization that can potentially tap different chemical reservoirs in the ice crust to be reflected in Europa’s surface composition. Table 1 lists specific compounds that have spectral features that MISE can detect to differentiate between the compositional signatures of the various formation mechanisms. Comparing the ice crystallinity of the plates of preexisting terrain with that of the surrounding terrains will provide insights into the thermal history of the chaos zones. To assess variability and potential reservoir differences, the composition of chaos terrain must be mapped at a variety of latitudes, longitudes, and spatial scales. Fortunately, chaos regions have been estimated to cover 20–40% of the surface of Europa (Collins 1981; Riley et al. 2000; Figueredo and Greeley 2004) and range in size from ∼1–100 km in diameter (Collins 1981; Riley et al. 2000; Spaun and Head 2001) making them easy to target. In general, *Galileo* Solid State Imager (SSI) regional data at resolutions of ∼300 m/pixel (Fig. 5b) provides sufficient spatial resolution to identify blocks, ridges, domes, and other geologic features.

#### Europa Geology

The compositional data collected by MISE will provide a history of tectonic processes on Europa, which is recorded in the numerous ridges, bands, and fractures that comprise the lineaments that characterize the Europa surface (Geissler et al. 1998; Greenburg et al. 1998; Head and Pappalardo 1999; Kattenhorn and Hurford 2009; Prockter et al. 2009; Kattenhorn and Procter 2014). These bands may have variable compositions, such as water ice crystallinity or variations in non-ice materials (Fig. 6) that would reflect their different formation mechanisms and/or relative ages, which may also be correlated with their morphology. Thus, comparing spectra collected over bands of differing ages (as inferred from cross-cutting relationships) will provide insight into the apparent brightening of bands with age (Pappalardo and Sullivan 1996). For example, observations by MISE should enable the determination of whether these trends reflect exposure to the Jovian radiation environment or an endogenic evolution of the emplaced material. These observations should also provide new information about the genesis of the dark material that flanks many ridges (Belton et al. 1996) (Fig. 5a, 25 m/pixel), constraining lateral variability in composition at spatial scales comparable to the ridges. As shown in Fig. 5, a study of Europa’s ridges and bands will provide information about processes active over a wide range of spatial scales.

#### Plumes

Observations by MISE can potentially be used to identify recent deposits of plume material on Europa’s surface, which may be apparent as small regions with distinct spectral and photometric parameters, thus indicating they disrupt or overlay global geologic or exogenous effects. For example, since Enceladus’ plume particles consist of highly crystalline water ice (Dhingra et al. 2017), fresh plume deposits on Europa could also contain more crystalline ice than found in other regions of Europa (cf. Postberg et al. 2018), potentially becoming more amorphous due to accumulation of structure damage from the bombardment of energetic ions in the Jovian magnetosphere. Such deposits could be identified based on spectral signatures in the various water-ice absorption bands (Mastrapa et al. 2009; Quick and Hedman 2020). Additionally, Hansen and McCord (2004) found that ice type on the three outer Galilean satellites correlated with the intensity of the radiation environment (charged particles with energies >100 keV) surrounding those moons. One hypothesis was that particle radiation converts crystalline-ice to amorphous-ice, as has been shown in the laboratory; thus, if the fresh ice from Europa’s putative plumes is crystalline-ice, as found for Enceladus, the amount of amorphous-ice in the plume deposit regions could serve as a coarse metric for the plume ice’s exposure time.

Detections of recent resurfacing from plume activity lead to pinpointing locations for the additional study of newly erupted material as well as potential sites for future landers and potential ocean explorers. HST data suggest that plumes of water vapor could be emerging from beneath Europa’s surface (Roth et al. 2014; Sparks et al. 2010; Jia et al. 2018; Paganini et al. 2020), sourced from the ocean or near-surface reservoirs (Howell and Pappalardo 2020; Vorburger and Wurz 2021). MISE should be able to observe the small particles of solid matter lofted above the surface by these plumes. Even though such particles have not yet been directly observed above Europa, particle-rich plumes are found above both Jupiter’s moon Io (Cook et al. 1979; Collins 1981; Zhang et al. 2004; Spencer et al. 1997, 2007; Geissler and McMillan 2008) and Saturn’s moon Enceladus (Dougherty et al. 2006; Hansen et al. 2006, 2011; Porco et al. 2006, 2014; Spitale and Porco 2007; Spencer et al. 2009; Viviano et al. 2014), and diffuse patches of fine-grain ice surrounding fissures on Europa’s surface could be created by fallen plume material (Fagents et al. 2000; Quick et al. 2013).

In principle, MISE could also directly detect plume particles if they can be viewed in the appropriate geometry and with adequate resolution. Many particles in Enceladus’ plume are in the micron size range (Hedman et al. 2009; Kempf et al. 2010). The particles in any Europan plume are likely to be similarly small and therefore forward scattering, so remote-sensing instruments will most likely detect these particles when the plumes are observed at high-phase angles (>160°). These high phase angles are also optimal for plume detection because they cause the limb and terminator to be close to each other, potentially allowing the lit plume material to be seen above a dark limb, minimizing stray-light contamination from the surface. In these situations, both the small size of the particles and the high phase angles suppress many diagnostic absorption bands (Hedman et al. 2009; Clark et al. 2019), but MISE should still be able to discern the strong 3-μm water-ice band and thus determine the grain size and composition of the particles in Europa’s plumes (Fig. 7). Spatially resolved, high phase angle spectra should also provide information about the launch velocities of the plume particles.

However, an important difference between the possible Europa plumes and the plume detected at Enceladus are their likely spatial extent. The Galilean satellites are much larger than Enceladus, and so plume material requires higher energy to reach the same heights and to spread as far as material ejected by Enceladus’ plume, a significant percentage of which goes into orbit and forms Saturn’s E ring (Hamilton and Burns 1994; Kempf et al. 2018). Eruption processes otherwise being equal, the plume surface deposits on Europa may have a smaller extent, which might make them easier to untangle from larger-scale trends due to exogenous processes and other global-scale water-cycles but would also require high spatial resolution compositional mapping to identify (Fagents et al. 2000; Quick et al. 2013; Quick and Hedman 2020).

#### Thermal Anomaly Detection

Given the geologically young, relatively crater free surface of Europa, with surface features characterized by fracturing and likely cryovolcanic features (Daubar et al. 2024, this collection), sites of active resurfacing could be detected through thermal emission. Such thermal anomalies would form at sites of active eruption and emplacement, including plumes, surface flows, evolving chaos areas, active vents along ridges, and pits and domes in the process of formation. These areas of recent activity might be mantled by unweathered, pristine materials that sample Europa’s interior. MISE would detect such thermal emission between 3 and 5 μm (Fagents 2004; Abramov and Spencer 2008) if the thermal anomaly filled a significant portion of the instrument field of view at a sufficiently high temperature. Newly erupted brines at ∼273 K, well above Europa’s peak background temperature (∼130 K) would initially cool quickly to ∼250 K in about 11 hours (Davies et al. 2010), but would take considerably longer (∼20 days) to reach 180 K, a temperature still above the Europa non-active background peak temperature and which thus could be detected by MISE, in particular on Europa’s nightside. Detection of such thermal features at multiple wavelengths could constrain Europa’s peak surface temperatures, and the temperature and area distribution, within the range of detection all of which would be diagnostic of how the material was physically erupted. Whether MISE detects a thermal anomaly depends on the temperature and area distribution within a pixel. For example, MISE could detect the thermal signature with a SNR ratio of 10 for an area at 190 K, if it filled 10% of the MISE field of view (Fig. 8). At close approach to Europa (for example, 50 km), this would be an area of only 16 m^2^.

## Instrument Description

### General Instrument Approach, Description, and Operation

MISE leverages a high heritage push-broom configuration that builds and improves upon a long litany of successful spaceborne imaging spectrometers, with heritage stemming directly from the Jet Propulsion Laboratory (JPL)-led Moon Mineralogy Mapper (M3) (Green et al. 2011) and the Johns Hopkins Applied Physics Laboratory (APL)-led Compact Reconnaissance Imaging Spectrometer for Mars (CRISM) (Murchie et al. 2007). Enabling new capabilities of MISE include the Dyson spectrometer approach, which is more compact and has higher throughput than the traditional Offner design, and a two-sided scan mirror that enables unrivaled inflight absolute and relative radiometric and wavelength calibration, as well as precisely controlling the push-brooming of the imaging slit. Additionally, “on-board” processing that is designed uniquely for the high radiation European environment, enables both mitigation of the deleterious radiation-induced signal excursions in individual detector pixels and combination of multiple image frames with the precisely controlled motion of the scan mirror, which will increase the signal to noise ratio (SNR) of collected data.

Key instrument parameters are summarized in Table 2. The instrument uses a cryogenically (mechanically) cooled HgCdTe detector provided by Teledyne to achieve the spectral range of 0.8 to 5 μm. A scan mirror controlled to a verified accuracy of tens of microradians enables consistent sampling of Europa’s surface in the along-track (spacecraft motion) direction over all anticipated surface velocities, as well as enabling in-flight calibration for ensuring the most accurate interpretation of the acquired spectra. Located on the instrument outside the vault, the Focal Plane Interface Electronics (FPIE) digitizes the signal from the MISE focal plane before sending them to the Data Processing Unit (DPU), which is located inside the vault and provides power control and software control for the instrument. A second electronics box, the Cryo-cooler Electronics Unit (CEU), provides control of the cryocooler. On-board processing by the DPU leverages the precise “spatial oversampling” capability of the scanner to both increase the SNR of each spectrum through the averaging of multiple spectra and the identification of anomalous signals due to radiation effects in the detector. A long-life, low-vibration “pulse-tube” cooler provided by Lockheed Martin mechanically cools the detector as well as the spectrometer, enabling the detector to operate at low noise. It also reduces the background signal from thermal emission by the instrument that would otherwise contribute to signal in the 3 to 5-μm region. The telescope is cooled passively with the edge of the telescope baffle acting as a radiator. The waste heat from the mechanical cooler is expelled to space via an upwards-facing radiator panel. The instrument is thermally isolated from the relatively warm deck of the spacecraft by titanium bipods, which also provide the rigidity to withstand vibrations during launch.

**Table 2:** Key Instrument Parameters

Instrument Parameter	Instrument Performance
Angular resolution	251 μrad
Angular field of regard	4.31° × 60°
Spectral range	800 nm to 5000 nm
Spectral sampling	10 nm
Spectral integration time	53.47 ms
Optical Parameters	
Focal length	119.3 mm
Slit width	30 μm
F number	f/1.4
Operating temperature of Focal Plane	83–87 K
Operating temperature of Spectrometer	84–92 K
Spectral Response Function (SRF) FWHM	1.32 pixels
Cross-track spatial Response Function (CRF)	1.26 pixels
Along-track spatial Response Function (ARF)	1.42 pixels
Spectral uniformity (in units of percentage of 30-μm pixels)	2.8%
Spatial uniformity (in units of percentage of 30-μm pixels)	3.5%
Detector	
Type	Teledyne Chroma HgCdTe
Array format	320 × 480 pixels
Pixel size	30 μm × 30 μm
Instrument dimensions	0.66 m × 0.63 m × 1.08 m
Instrument mass	∼65 kg
Instrument power (max, nominal, standby)	∼60 W (operating)

### Focal Plane and Signal Chain

The MISE Focal Plane Array (FPA) uses an anti-reflectively coated HgCdTe detector layer that is hybridized to a silicon ReadOut Integrated Circuit. The bandgap of the HgCdTe is tuned for a cutoff of 5.2 μm to allow high sensitivity across the near-infrared (NIR) and into the mid-infrared. The detector operates at 85 K to minimize dark current and its associated noise. The FPA is shielded from radiation by tantalum and aluminum housings.

**Fig. 1:**
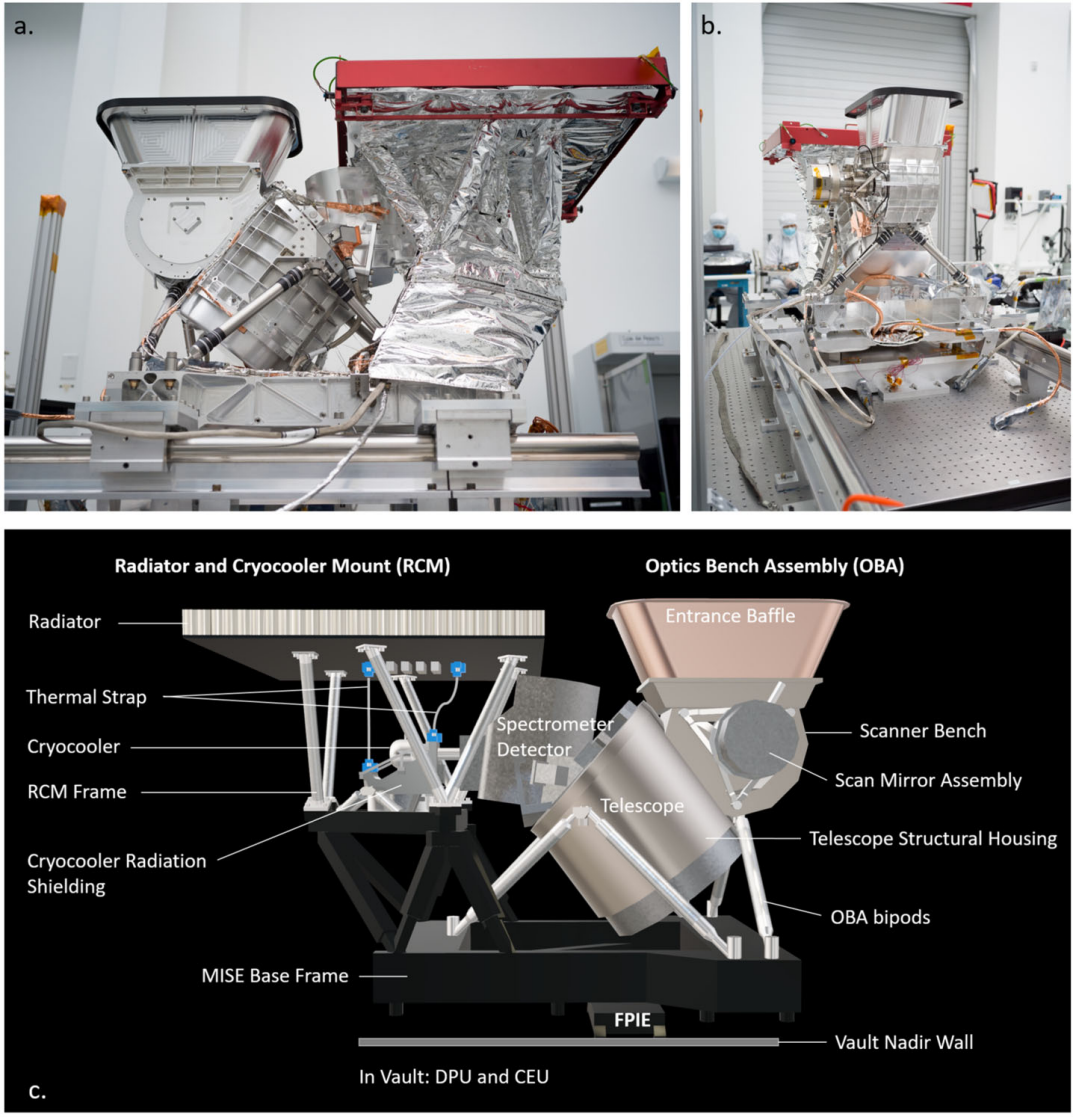
(a) The MISE instrument in a clean room at JPL in May 2023. The Entrance Baffle and Radiator have temporary protective covers. (b) A view of MISE showing the Scan Mirror Assembly. (c) The main components of MISE. The Optical Bench Assembly (OBA) consists of the OBA bipods, telescope, spectrometer detector (Teledyne) and the Scan Mirror Assembly (APL). The Radiator and Cryocooler Mount (RCM) consists of a cryocooler (which is radiation-shielded), radiator, and a Thermal Strap. The OBA and RCM are mounted on the MISE Base Frame. Figure 1a shows the RCM sheathed in mylar for electronic noise protection. The Base Frame is mounted on the Europa Clipper vault nadir wall. The MISE Data Processing Unit (DPU) and Cryocooler Electronics Unit (CEU) are in the spacecraft vault. The Focal Plane Integrated Electronics (FPIE) are mounted on the outside of the vault. Photos by Ryan Lannom

The MISE FPA outputs four analog channels, each addressing 80 spatial columns and 480 spectral rows, for a total of 320 spatial columns. These are driven through a rigid-flexible circuit internal to the spectrometer housing and then external cables into the Focal Plane Interface Electronics (FPIE) box. The FPIE performs the analog-to-digital conversion of the FPA data and provides the FPA with regulated power and control signals. The FPIE is situated on an external face of the spacecraft vault (Fig. 1) between the spectrometer and the Data Processing Unit (DPU). The DPU is in the vault. Due to its external location, the FPIE housing is made from tungsten copper.

**Fig. 2:**
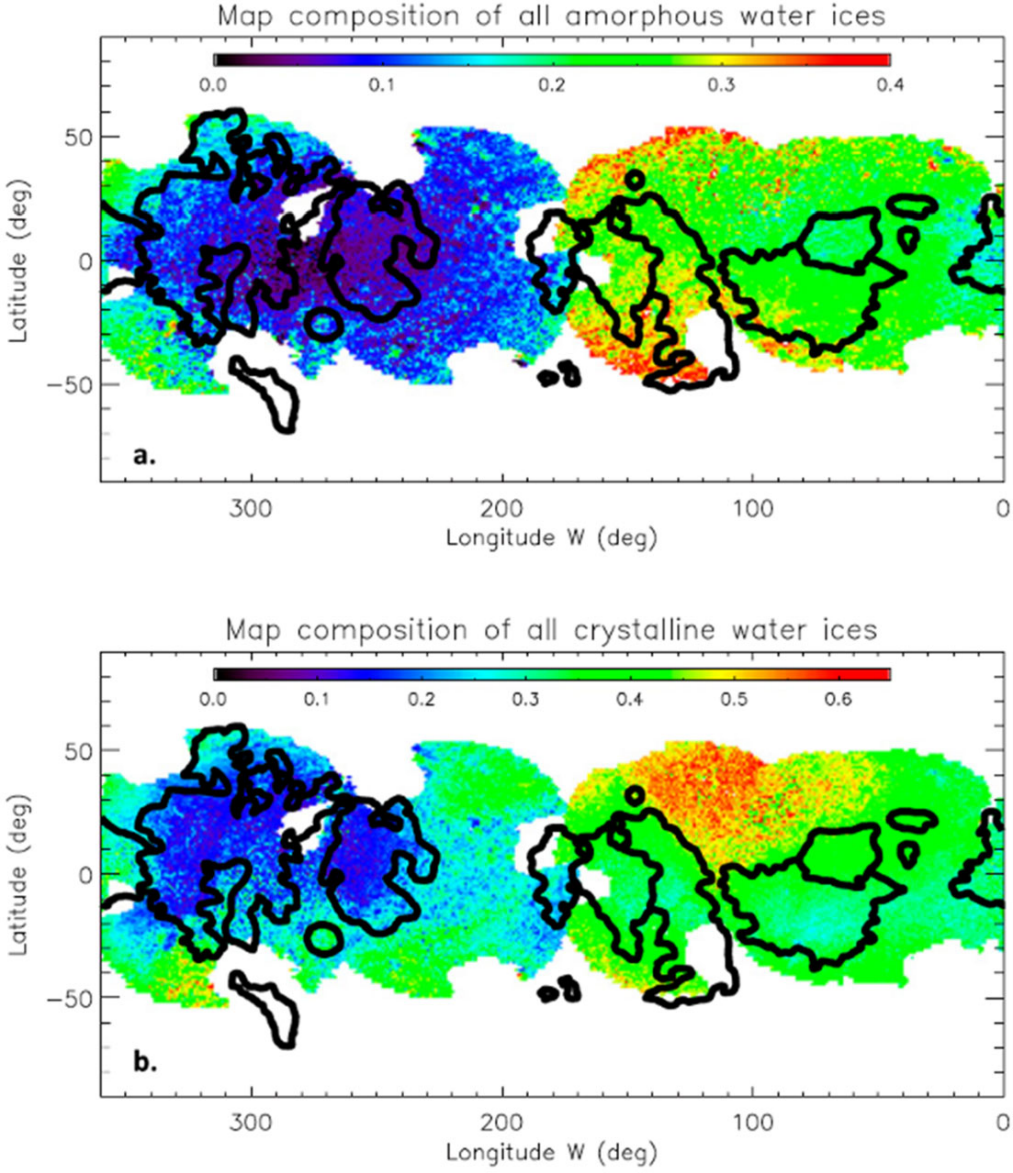
The distribution of amorphous water ices (top) and crystalline water ices (bottom) on Europa’s surface, as derived from ground-based telescope data (from Ligier et al. 2016). By summing the fraction (see color bar) of both states of ice from the two panels, it is possible to estimate the amount of total water ice in the top layer of Europa’s surface. Most notably, the trailing apex, which faces into the plasma ram, is relatively depleted of water ice. See also Brown and Hand (2013). MISE will extensively map the type and state of ice across Europa

The maximum frame rate from the FPA is 18.2 Hz, and each pixel is digitized to a depth of 14 bits. The resulting raw data rate is 38 Mbits/s, with a maximum possible integration time of 54.48 ms. An integration time of 53.47 ms is planned for flight to match the instrument radiation analysis to date.

### Optical Design

MISE is designed to operate at f/1.4 over 0.8 to 5 μm with 10 nm spectral sampling and 251 μrad/pixel spatial sampling with high spectral and spatial resolution and uniformity. In addition to high throughput and uniformity, the spectrometer design is driven by the requirement for heavy shielding of the detector, which demands a miniature design to reduce shielding mass and dictates specific geometry constraints for the optical path. MISE can accommodate the full spectrum with a single focal plane and a unique, tailored grating efficiency. Meanwhile, a displaced slit design minimizes filter reflection ghosts. The MISE optical bench assembly and optical ray-trace is shown in Fig. 9.

The MISE f/1.4 fore-optics (Fig. 9a) consist of a double-sided scan mirror and a two-mirror telescope with a 4.3° field of view in the cross-track direction. The scan mirror operates in the along-track direction, providing a 60° field of regard by rotating its specular side ±15° mechanically (±30° of surface coverage). The opposite side is a diffuse gold surface used for inflight flat field and radiometric calibration. The mirror can also be closed for dark field calibration. The ∼120 mm focal length telescope consists of two gold-coated aluminum off-axis aspheric mirrors and focuses to a 9 mm long, 30 μm wide instrument slit. On the other side of the slit, the Dyson spectrometer (Fig. 9b) consists of CaF_2_ transmissive optics and a concave diffraction grating fabricated at JPL. The MISE optical design performance meets the required specifications to achieve the science planned for the instrument while accommodating the required shielding and geometry constraints. To meet the standards of high-performance imaging spectroscopy, required non-uniformity values are <10% of a pixel for geometric non-uniformities, with spectral and spatial response function FWHM ≤ 1.5 and 1.7 times the sampling interval, respectively.

### Thermal Design

The MISE thermal control architecture consists of both passive and active elements. The telescope and scan mirror temperature are passively cooled. The focal plane and spectrometer require active cooling to the 83–92 K range. Spectrometer and detector temperatures are maintained by the cryocooler. Cryocooler power regulation and control is provided by the Cryocooler Electronics Unit (CEU), which resides in the spacecraft vault.

The thermal design (Fig. 10) provides five temperature zones external to the vault at 83−84 K (FPA), 87−90 K (spectrometer), 135−158 K (telescope), 145−150 K (cryocooler survival), 195−227 K (cryocooler operational), and 273−323 K (FPIE/CEU/DPU). The Radiator Cryocooler Mount (RCM) (Figs. 1 and 10) supports the cryocooler, the thermal strap (comprising two pyrolytic graphite straps, or PGS), and the cryocooler radiator. The cryocooler and radiator are thermally isolated because they are warmer than the rest of the instrument during operation. The detector and spectrometer are cooled by the cryocooler to 83–87 K and 84–92 K, respectively. The thermal strap transports waste heat from the cryocooler to the radiator surface and provides the target temperatures for the detector and spectrometer with a single temperature control point at the cryocooler cold tip, which is thermally coupled to the detector and spectrometer and is temperature controlled to a few Kelvin lower to provide cooling. The thermal strap, with two connecting legs, has the thermal conductance required to remove the detector and spectrometer heat loads. The thermal strap end fittings are aluminum with copper braids in the mid-section to provide required heat transport between the detector, spectrometer, and cryocooler cold tip.

The telescope assembly is passively cooled to 150 K via the aperture baffle and small radiator attached to its perimeter. The telescope has three main heat loads: 1) scan motor power dissipation; 2) spacecraft radiative and conductive via mounts and cabling with shielding; and 3) external heat loads from the Sun and Europa. The Optical Bench Assembly (OBA; Fig. 1) is constructed from aluminum and is mounted on the instrument base frame structure (Fig. 1) with low conductance titanium struts. The FPIE is mounted near the optical bench directly on the vault surface. FPIE temperature is controlled by the vault interface via the spacecraft fluid loop heat rejection system. The DPU and CEU electronics chassis inside the vault are also cooled via the spacecraft fluid loop heat rejection system. The DPU controls the instrument and interfaces with the spacecraft. Cryocooler power regulation and control is provided by the CEU that resides in the spacecraft vault. CEU command and telemetry is managed by the DPU.

Thermal control elements include multi-layer insulation (MLI) blankets and surface coatings. The latter manage the transfer of waste energy from sources through structures and ultimately to radiating surfaces including a dedicated radiator. Active thermal control employs close-loop on/off heater control with settable temperature setpoints and deadband control.

The overall instrument thermal design focuses on the three low temperature thermal control zones: 1) focal plane temperature zone near 85 K for the detector, and spectrometer optics, 2) optical bench near 150 K for the telescope optics, and 3) cryocooler temperature zone at 220 K with its radiator. The FPIE is mounted directly on the vault surface underneath the OBA to minimize the cable distance to the detector. The 85-K zone is provided by means of a small long-life, flexure-bearing, mechanical pulse-tube cryocooler. The cryocooler cold tip temperature is close-loop controlled by the CEU using a proportional integral (PI) loop with the appropriate parameters. The cryocooler is designed to operate at 210–240 K to increase thermodynamic efficiency and reduce cooler power required. This cooler was also designed to survive very cold temperatures when not operating. In survival mode, the cooler temperature drops and is controlled at 145–150 K with a 5 W survival heater. The cooler underwent radiation testing to 500 krad and three-times life testing. It has been extensively characterized at JPL producing sufficient data to create a multi-variable performance model for use in thermal vacuum testing and flight predictions. The 150 K zone is provided by means of passive radiative cooling mainly via the instrument aperture and its baffle radiator. The 220 K zone is achieved by using a radiator coupled to the cryocooler via two high conductance pyrolytic graphite straps. Figure 10 also shows the temperature zones for different instrument components.

The RCM is a standalone thermal-mechanical assembly that provides cryogenic cooling for the instrument (Fig. 1). The Z93C55 white conductive paint used on the radiator was extensively tested for performance under the thermal and radiation environments expected for this mission (McKinley et al. 2021). The radiator is a lightweight structure with aluminum honeycomb and thin facesheets. To provide the structural stiffness required for the radiator to meet the modal frequency requirements, its core thickness was increased to 3.8 cm (including the facesheets). This increase in thickness results in lower thermal conductance through the radiator, resulting in a small increase in cooler sink operating temperature. The through-thickness conductance of the radiator was measured as a function of temperature and was used in the model for all the thermal analyses.

Parasitic heat leak to the 85 K zone is minimized by means of a low thermal conductance detector Moore mount design using small diameter titanium rods. Gold-coated surfaces are utilized to reduce radiation heat transfer to the detector, spectrometer, and its flexible gold-coated Kapton ribbon cable. The estimated total heat leak, including parasitic, to the cryocooler cold tip at steady-state is ∼450 mW at 80 K.

#### Cryocooler

In operating mode, the cryocooler is powered on to provide the heat lift required to maintain the FPA and spectrometer within allowable flight temperature limits. When the cryocooler is first powered on, it operates at maximum voltage with approximately 25 W of input power. When it reaches its cold tip setpoint, the cooler input power drops to less than 20 W. Because the cryocooler waste heat emitted to space via the radiator is not constant, the cooler sink temperature will exhibit a short duration peak before it will stabilize at a constant temperature (Fig. 10). The hot case results were initially baselined from a Venus flyby transit scenario with full sun exposure on the radiator for up to 80 minutes. The cold case is a steady-state scenario with the radiator staring at cold deep space.

#### Decontamination

Cryogenic instruments like MISE are required to undergo an extensive outgassing period immediately after launch to dissipate residual water moisture in the instrument and surrounding spacecraft structure. Additionally, higher molecular weight outgassing products will tend to collect on cold surfaces, so this initial outgassing period is critically important to the long-term performance of MISE. The abundance of water moisture on spacecraft systems at ambient conditions is a persistent concern for cryogenic systems, independent of the high temperature vacuum bakeouts done at the component, instrument, and spacecraft levels. High-temperature bakeouts expel high molecular weight volatiles with high activation energies, requiring bakeout temperatures in excess of 323 K. Once these clean surfaces are exposed to ambient conditions prior to flight, water in great abundance will be re-absorbed fairly quickly on all surfaces and in particular on composites and insulating blankets. The time constant for the reabsorption of water on these surfaces is in the order of a few hours.

Water ice will deposit on cryogenic surfaces at temperatures in the order of 155 K or less at typical partial vapor pressures surrounding spacecraft in space in the order of 10^−6^ torr. In space, decontamination temperatures in excess of 200 K will typically suffice to sublimate any build up on cryogenic surfaces. Thin water layers of the order of a few μm thick lead to increased surface effective emittance, resulting in increased radiative parasitic heat loads on cold cryogenic surfaces. The accumulation rate will show up as increased cryogenic heat loads on the cryocooler and detector loss throughput signal. MISE can be placed, if required, in decontamination mode after data acquisition has ended at the end of a Europa encounter sequence.

### Scan System

#### Precision Scan Mechanism

The MISE Scan System (Fig. 11) provides precision control and pointing of a two-sided, gold-coated mirror. The design is driven by optical and mechanical pointing requirements, with the former largely determining the mirror parameters and the latter constraining the mechanism and electronics design. The resulting design consists of a cantilevered, lightweight, two-sided AlBeMET (Aluminum-Beryllium metal matrix) mirror with a diffusely reflecting gold coating on one side and specular gold on the other. The mirror is supported by an all-steel 440C angular contact bearing complement, arranged in back-to-back configuration with a soft preload of 66.7 N provided by a wave spring. The cages and races of the bearing are coated in MoS_2_ for cryogenic dry lubrication. The bearings are emplaced on a MoST (MoS + Titanium) coated Ti shaft. The soft preload of the system requires that one of the bearing races slides a small amount to accommodate for the geometric change in the bearing contact angle as the system temperature changes. The MoST shaft coating provides lubrication for this motion. The shaft of the MISE scanner is driven by a zero-cogging brushless DC motor (BLDC). Readout of the angular position is provided by an Inductosyn-type encoder manufactured by Ruhle. Electrical signals are required on the motional part of the scanner to read the temperature sensor on the scan mirror and to drive the rotor of the Inductosyn encoder. This electrical connection is provided by a Kapton flex cable with Arlon 85 boards and associated wiring. The motion of the scanner is restricted to 290 degrees by a hard stop that maintains winding of this flexible cable. Temperature monitoring on the mirror and of the motor housing is provided by platinum resistive thermometers. The materials chosen minimize coefficient of thermal expansion (CTE) mismatches while providing the needed mechanical and structural performance.

The pointing performance requirements for the MISE Scan System (scanner) are largely quantified by three specifications: the knowledge (the uncertainty of measured positions of the scan mirror), the accuracy (the difference between the actual angle and commanded angle), and the stability (which is the same as the accuracy except any slow or static offset is not included). The initial pointing requirements for the scanner were 20, 60, and 125 microradians 3-sigma for the knowledge, stability, and accuracy, respectively. These initial pointing requirements motivated many engineering choices in the mechanical and electrical designs. As the complete instrument design evolved and the error budgets became clearer, these were able to be relaxed to 50, 100, and 125 microradians, respectively. The control system has been designed to cancel disturbances to the motion of the scanner so as to hold the scanner at the desired angle. Given that an unavoidable disturbance to a system of this type is the torque noise of the bearing that supports the motion of the scanner, then in a relatively quiescent environment with sufficiently well-designed electronics, this bearing noise becomes the primary disturbance/noise that the control system will compensate for. The MISE scanner provides piecewise linear approximations of the input scan curves. This reduces the core control requirement to provide the best possible constant velocity motion at a wide range of velocities.

There is interplay between the moment of inertia of the scanner and the torque applied by the motor and the bearing noise. For a fixed bearing torque noise, a higher moment of inertia of the scanner will reduce the velocity change induced by that torque and thus reduce the velocity error. Increasing the moment of inertia of the scanner requires the applied torques from the motor to increase proportionally to produce the same slew rate. The moment of inertia is therefore tied to the power consumption of the mechanism and the size of the motor. The goal of the scanner design was to minimize the stability error in the scan system, which depends on the scaling of the torque noise of the bearings in ratio to the noise levels of the encoder and the motor driver. The torque noise of the bearing is linearly proportional to the preload, as friction is directly proportional to the normal force applied. To minimize the torque noise of the bearing, the MISE scanner uses a soft preload to set the minimum acceptable preload for launch to maximize the scanner performance during the mission. The motor used in the MISE scanner is of the zero-cogging type for minimizing the variation in torque needed to provide smooth motion. Thus, as the cogging is smooth and does not greatly increase the peak torque requirements, cogging levels are not a critical driver of performance. The control electronics were optimized and thus are only a small contributor to noise.

The encoder used in the scanner is an Inductosyn, a 128/127 sector absolute type encoder on a titanium substrate. This is a segmented variable multiphase transformer where the ratio of the amplitudes of multiple quadratures indicate the position of the encoder. An inductosyn was chosen for this system primarily because of the high-radiation and cold operating environment of the scanner. In this scheme, the absolute angle is the sector and the position within that sector. The sector is inferred by the difference in the current sector position on the 128 and 127 tracks. In less harsh environments, other types of encoders, such as optical encoders, may offer superior performance. The connection to the encoder and temperature sensors on the motional part of the scanner is provided by a flexible Kapton cable. This flexible link’s bending force adds to the system overall torque and so was intentionally minimized. In the MISE scanner, we estimate this element required less than 0.0014 N-m of torque to bend at nominally 100 K. This cable touches itself and other surfaces in the scanner housing as it twists, such contact creates friction which acts as a disturbance. In the MISE scanner, this friction appears to be much less than that of the bearing in all configurations.

#### Control Methodology (Inductosyn) and Pointing Approach and Performance

The MISE scanner provides piecewise linear approximations of scan curves within a velocity bound of 10°/sec and quantization limit of 16 bit. To provide this, the scanner motion must be smooth and constant, which is accomplished through active control of the scan mirror’s angular position using a proportional-integral-velocity (PIV) control loop (Fig. 12). The control loop attempts to correct an error, defined as the difference between the desired position and the current position, via the drive currents applied to the motor. The driver for the motor is a two-phase linear current source. This approach reduces the noise of the electric torque applied while maximizing the precision and minimizing electromagnetic interference (EMI), while sacrificing some power efficiency in the DPU. The motor in the MISE scanner can also be driven by a higher-torque, pulse-width modulated (PWM) driver that was provided to release the system from the descoped launch lock. It operates in voltage mode, and the torque is a function of the motor temperature. This mode is available via macro control implemented in the flight software to overcome possible sticking events. Control of the scanner angle depends on the bandwidth of the control loop and thus the update rate of the encoder. The update rate of the Inductosyn is low compared to other encoder technologies. This low bandwidth is largely due to the low drive frequency (10 kHz) used in this component to sense the position.

The Inductosyn encoder provides a stable mapping of the rotation of the shaft to code. The systematic errors in this encoder depend on the mounting of the rotor and stator with respect to each other but generally were observed to be of the order of 100 microradians peak to peak. This systematic error is reduced in the MISE scanner by calibrating the Inductosyn against a better encoder. For the flight unit, an optical encoder was used as the transfer standard. An autocollimator-based method was also used with light reflected off the specular side of the mirror. The calibration data are applied to the system via a linearly interpolated look-up table (LUT). Room-temperature testing showed that after removal of this systematic error, the system was generally repeatable to within 15 microradians, 3-sigma with respect to the transfer standard for motions in the calibrated angular region. Assuming a linear trend, one extrapolates an error of ∼13 microradians at operating temperature. As this calibration method corrects for systematic error there is no way to observe its drift over time without an external reference. For this reason, we carried out a careful analysis of the electronics that drive and sense the angle to budget for a potential increase of 26 microradians in the knowledge error over the life of the mission, accounting for a constant random error of 6.3 microradians and an estimated drift in the calibration of ∼11.5 microradians.

#### Scan Mirror

Both sides of the mirror (Fig. 11) have optical surfaces: a specular side and a diffuse reflective side. The specular side is used for science measurements and those calibration measurements that require or can utilize imaging: geometric calibration using stars and limb views of extended sources, and wavelength calibration on known sources such as stars and possibly Jupiter’s atmosphere. The diffuse surface enables conducting in-flight radiometric and wavelength calibration using the Sun and flat fielding with any source that is sufficiently bright and uniform including the Sun. This eliminated the need for an internal calibration source and furthermore ensures calibration is conducted with the same source and optical path as science measurements. The mirror can also be closed to obtain a ‘dark’ or instrument thermal background calibration. Closed position is with the diffuse side facing out, to maintain the cleanliness of the specular side when not in operation.

The environmental and optical requirements (Table 3) greatly influenced the mechanical, structural, and optical design of the two-sided mirror. To survive the launch loads, the mirror was lightweighted first by use of AlBeMET as the substrate and second by use of EDM to cut out a triangular pattern of ribs running through the mirror. A neck was used to minimize the effect of bolt preload on the optical flatness. The diffuse side of the AlBeMET substrate was sandblasted as a final step in the AlBeMET machining process, then both sides of the mirror were coated with Nickel. The specular side was polished and then both sides were coated with an unprotected gold coating, thus achieving a purely metallic mirror capable of surviving and minimizing degradation in the Jovian radiation environment.

**Table 3:** Relevant scanner performance requirements

Parameter	Requirements
Pointing accuracy	125 μrad, 3-*σ*
Pointing stability	100 μrad, 3-*σ*, over 1.08 sec
Reflectivity of the specular side	> 95% IR, >93% NIR
Axis of rotation knowledge error	<50 μrad, 3-*σ*
Specular variation over scan angles	< 3% all wavelengths

Regarding the diffusivity of the calibration surface, throughput for operational calibration angles sufficiently close to that of a Lambertian reflector were required to reflect the appropriate amount of sunlight into the slit to obtain adequate SNR over the entire spectral range, without approaching detector saturation. A perfectly Lambertian surface is not required and was not obtained. The surface also needs to be devoid of spectral features over the 0.8–5 mm spectral sensitivity range of the instrument. Several different ‘roughening’ techniques were explored including plasma deposition and sandblasting, for which multiple techniques and sand grits were experimented with, by the manufacturer, General Dynamics. The end performance of the diffuse side is shown in Fig. 13, which compares the bidirectional reflectance distribution function (BRDF) of the diffuse surface to that of a Lambertian reflector. The approximate bounds of the operational range (defined by sufficient reflected solar illumination) are depicted by the green box. As can be seen from the data in this figure, the surface has negligible specular components out to 1.5 μm at ∼70° angle of incidence (Fig. 13); even at 3.6 μm the specular component is minimal. Furthermore, and essential, the functional shape of the BRDF is sufficiently independent of wavelength out to at least 3.6 μm, to enable calibration at all wavelengths in a single measurement at one angle of incidence (AOI). The magnitude of the BRDF is between ∼1 and 10% and is obtained with the mirror at ∼ 10 to 50° solar incidence angle are depicted by the green box.

### Data Processing Unit (DPU)

The MISE Data Processing Unit (DPU) is a 5-slice electronics box that resides in the spacecraft vault. The DPU has four main functions:

1. Provide power for other instrument components, including the detector electronics, cryo-cooler control electronics, and scanner via the Low Voltage Power Supply (LVPS). This is composed of two boards.
2. Acquire, store, and process spectrometer data. This is performed on the Focal Plane Memory Card (FPMC).
3. Precision control of the scan mirror for science and calibration data acquisition and control of MISE heaters and PRTs (thermometers). This is performed by the Interface Card (IFC).
4. House the flight software and provide general command and control of the MISE instrument as well as the spacecraft interface. This is performed by the Processor Card (PC).

#### Low Voltage Power Supply

The low voltage power supply for MISE is comprised of 2 boards or slices, A and B, in the larger DPU. Forward converters provide the main isolated secondary power supplies. Slice A contains the power rails needed for the FPIE and related cryocooler electronics and slice B provides the power needed for the IFC, the PC and the scan motor. Additional low voltage rails are provided with buck converters that follow the isolated forward stage. Linear post regulation of the switch mode stage is provided for rails that required lower ripple requirements. For the FPIE this post-regulation was implemented on LVPS A while the IFC card performs this function itself.

#### Focal Plane Memory Card

The primary function for the Focal Plane Memory Card is to store the raw data from the focal plane array as well as any compressed data and processed data. The FPMC also performs any data analyses (see Sect. 5). Due to the high radiation levels in the Jovian environment, spectrometer data are taken at relatively short integration times that are, generally, individually inadequate to attain the required SNR. The data are stored in the on the FPMC during flybys as raw data for post-processing and frame aggregation during the time between flybys. These analyses are the most computationally intensive and complex activities performed by the FPMC, both in terms of bandwidth and complexity. As such this slice contains the largest FPGA in the DPU for enabling hardware defined processing that, through inspection and analysis, compresses oversampled raw data into higher SNR, radiation-mitigated, data.

#### Interface Card

The interface card (IFC) of the DPU supports the motion control of the scanner, provides scientific temperature measurements and control of heaters. This slice of the DPU contains an FPGA that abstracts these inputs and outputs into a register file that is read/written by the processor card at a 1 kHz update rate.

The IFC reads the angle of the Inductosyn via two Resolver to Digital Converter (RDC) channels one for the 128-sector track and one for the 127-sector track. The frontend circuits that drive these two RDCs are carefully trimmed and matched with low-drift components. Mismatch in the balance of the sine and cosine readout of the Inductosyn would become a systematic angular error (Hanselman 1990). Given the long duration of this mission, these drifts were minimized as far as technically possible. For the RDCs to read the Inductosyn, a sine-wave drive must be provided to the rotor of the encoder. This is provided by direct digital synthesis in the IFC FPGA of the required tone. This output is then put through an active filter before driving the rotor of the Inductosyn. Higher-order harmonics introduce error terms in the angular readout. By filtering the drive signal carefully, the IFC design enables these terms to be negligible.

The IFC provides two types of drive circuits for the scanner motor: a linear current source driver for normal operation and a voltage mode H-Bridge PWM driver for operating with more torque but at lower precision. Both drivers can address the primary and redundant windings on the scan motor. The linear current is the primary driver used to collect science data. The linear current source has a resolution of 12-bits and a 10–90% step time of nominally 0.2 ms. This step response time, like the update rate of the Inductosyn angle, limits the bandwidth of the overall control system. The PWM driver provides a higher torque, lower precision (being controlled with a 7-bit precision) pointing if scanner friction exceeds the 200% torque margin of the linear motor drivers. There are four H-Bridges to address the two phases and primary and redundant windings. However, there are only two linear current drivers that can be switched to the needed phases. The two drive methods are selected via the configuration of the H-bridge and a latching relay.

The heaters driven by the IFC are resistive and control of these components is provided by a simple high-side switch that is modulated via the register file to achieve the average heating needed. The temperature measurements are provided by a multiplex constant current four-wire measurement. Two of the multiplexer channels are used to measure two low-drift reference resistors on the IFC. The temperature sensors used are platinum resistive thermometers (1000-ohm type). The two reference measurements make up the measurement radiometry (enabling removal of the offset/slope errors) to within a small channel-to-channel mismatch factor that is proportional to the input bias current of the instrumentation amplifier used to sense the voltage on the sensor.

#### Processor Card

The processor card interfaces with the spacecraft computer via a redundant SpaceWire interface. It controls the complete operation of the MISE instrument by receiving commands from the spacecraft, decoding these commands, and controlling the rest of the instrument via the DPU slices. In return, the instrument collects telemetry data and sends that back to the spacecraft through the SpaceWire interface. Images that are collected and processed by the FPMC are sent to the processor card where the data are packetized and transferred to the spacecraft at a rate of 20 Mbits/s. Various housekeeping data are also collected from different parts of the instrument, which are also packetized and sent to the spacecraft. The processor card also provides the control processing for the scan mirror. It implements the PIV control loop discussed in the Sect. 3.5.2 and updates the IFC at a rate of 1 kHz to maintain control of the mirror.

### Other Design Considerations

The high-radiation Europa environment and need for compatibility with the other science instruments and systems on Europa Clipper introduced additional design considerations for MISE. The radiation environment, in particular, was a major driver of the MISE design. Electronic parts were limited to radiation-hardened parts which had passed the Europa Clipper parts analysis. MISE electronics assemblies are therefore shielded to 300 krad. Transport analysis was used to identify locations and parts that required spot shielding and was added as necessary. The focal plane and spectrometer are encased in ∼10 mm of Tantalum and 8 mm of Aluminum to keep the number of radiation hits at a level that could be managed by the onboard processing unit. This shielding resulted in a predicted Total Ionizing Dose (TID) of 15 krad at the focal plane and 20 krad for the spectrometer (Radiation Design Margin (RDM) of 2). These low dosages also limit potential changes in the optical properties of the materials in the spectrometer. The gold mirrors in the telescope, which receive doses of radiation between 10–22 Mrad (TID, RDM=2), were uncoated to minimize the risk of any performance degradation from these higher radiation dosages.

Susceptibility to internal electrostatic discharge (IESD) limited the materials that could be used in MISE construction, especially for components outside the spacecraft vault. All paint applied to MISE is conductive and approved as consistent with the Europa Clipper IESD plan. Designs were independently analyzed to ensure no adverse IESD effects on MISE hardware or the Europa Clipper flight system.

Contamination Control for MISE is driven both by the instrument being a cryogenic optical device capable of cold trapping materials and by ensuring outgassing rates that do not impact the other science instruments on Europa Clipper. For optical cleanliness, MISE follows standard clean room practices for optical instruments. All materials have been reviewed for outgassing and selected with the outgassing needs in mind. Cables, electronics boxes, and other parts of the instrument have undergone bakeouts. Surfaces and parts are precision-cleaned prior to assembly. MISE cleanliness is continually monitored, and cleaning of exposed surfaces is performed as appropriate. When not required to be uncovered, the telescope baffle and the radiator have “remove before flight” covers to protect interior surfaces. The outgassing rate from MISE will be verified via Cryogenic Quartz Crystal Microbalance (CQCM) measurements prior to delivery for integration with the spacecraft bus. Due to the more stringent contamination control requirements, planetary protection requirements are expected to be met via the MISE Contamination Control plan and will also be documented at time of delivery.

### Calibration

Calibration quantifies the spectral, spatial, uniformity, and radiometric properties of the imaging spectrometer system. This allows translation from the recorded digital numbers on the sensor to a quantitative spectral radiance measurement with corresponding uncertainty estimates, which is crucial for the science goals of MISE. The MISE calibration approach builds on a rich history of Solar System and Earth-imaging spectrometers including NIMS, VIMS, CRISM (Murchie et al. 2007), M3 (Green et al. 2011), the Advanced Responsive Tactically-Effective Military Imaging Spectrometer (ARTEMIS) (Lockwood et al. 2008), and the Earth surface Mineral dust source InvesTigation (EMIT) (Thompson et al. 2024). An example of the translation from raw recorded signal to quantitative measurements is shown in Fig. 14.

Many measurements of the MISE instrument were collected with a range of sources to characterize its spectral, spatial, uniformity, and radiometric characteristics (Table 4). These measurements and preliminary results are described briefly below. An additional publication is planned that will contain the final pre-flight calibration information for MISE. Further validation and refinement of the MISE calibration files and characteristics are expected based on in-flight observations. These planned measurements include views of the Sun, other stars, Jupiter, and other objects in the Jovian system, including Io. These validation measurements can be used to reestablish the MISE calibration parameters if discrepancies are discovered in-flight, which is a standard practice for imaging spectrometers, including M3 and EMIT. In-flight calibration details are discussed in Sect. 4.4.

**Table 4:** MISE calibration files required to deliver calibrated measurements to achieve the science objectives

Name	Interpretation	Description
Average Spectral Calibration ASCII File	Channel, center wavelength, and FWHM in microns	Spectral calibration and uncertainty (nanometers)
Spectral Calibration Array	Center wavelength in first channel, uncertainty in second, FWHM in third, uncertainty in FWHM in fourth	Spectral calibration and uncertainty (nanometers) for all pixel elements
Spatial Cross-Track Response Function	Function that describes the cross-track response	Describes the convolution of the cross-track signal in the spectrum
Spatial Along-Track Response Function	Function that describes the along-track response	Describes the convolution of the along-track signal in the spectrum
Spectral Stray Light Factors	Correction matrices as in Chapman et al. (2019)	Brings response functions towards Gaussian
Spatial Stray Light Factors	Correction matrices as in Chapman et al. (2019)	Brings response functions towards Gaussian
Bad Pixel Mask	Zero indicates good pixels. <1 indicates bad pixels and number of contiguous bad pixels in the spectrum. >1 indicated row and column masked pixels	From laboratory calibration measurements. Updated as needed
Radiometric Dark Level	Offset value to be subtracted from each element before radiometric analysis	From un-illuminated portion of orbit and masked detector elements
Radiometric Calibration Coefficients (SWIR/MWIR)	Channel, μW/cm^2^/nm/sr/DN, and one-sigma uncertainty	Maps digital numbers (DN) to radiance
Radiometric Flat Field	Small relative radiometric corrections that refine the RCC-predicted radiance value. Should be close to unity. With uncertainties in second channel	Fine correction of radiometric coefficient across pixel elements
Linearity Map	Mapping from measured dark-subtracted digital numbers to ideal linear digital numbers	Linearity correction for all FPA elements
Spatial Camera model	The “look direction” and vertical iFOV of each cross-track element. Values contain Pitch, Roll, Yaw, Lateral iFOV FWHM (milliradian), Lateral Shape parameter (unused), Vertical iFOV FWHM (milliradian), Vertical Shape parameter (unused)	In concert with position, attitude, and scan mirror data, the camera model allows projection of the measured spectra on to the surface target

#### Spectral and Spatial Calibration

Throughout the alignment and calibration of the instrument, the overall optical performance is routinely assessed by measuring the spectral, spatial cross-track, and spatial along-track response functions. These measurements reveal the spectrometer resolution and uniformity in the three data cube dimensions.

The spectral response function (SRF) describes the spectral response of the instrument for each detector pixel. It is measured by illuminating the instrument with a monochromator at different spatial field locations. A Gaussian curve is fit to the intensity profile as the monochromator wavelength changes, giving the spectral FWHM for each channel. The mapping from channel to wavelength is determined by illuminating the instrument with four laser lines at 912.4 nm, 1900.2 nm, 3392.3 nm, and 4678.8 nm. Each wavelength is mapped to a row channel across the cross-track columns. The wavelength-channel mapping for the remaining wavelengths is extrapolated from these measured lines by fitting the designed spectral sampling curve to the measured points. The designed spectral sampling is near 10 nm across the detector but varies slightly from 10 nm due to refractive index effects. The scaling factor used to fit the measurements to the design curve is close to unity, showing that our measured laser wavelength-channel mappings match the designed spectral sampling curve well. We take the mean across spatial pixels to determine the final average wavelength-channel calibrated mapping. Figure 15 shows the average spectral calibration, including scatter-alignment rows at the top and bottom of the detector, as well as masked rows at the bottom of the detector.

The cross-track response function (CRF) describes the spatial response of each pixel in the cross-track dimension of the detector array. It is measured by illuminating the instrument with a spatially narrow broadband source that is scanned cross-track across the detector array, giving the CRF centroid and FWHM. Similarly, the along-track response function (ARF) describes the along-track spatial response of the instrument for each pixel as the instrument scans in time, which is equivalent to the second spatial dimension for a push-broom spectrometer. To measure the ARF, an along-track spatially narrow broadband source illuminates the entire cross-track spatial dimension of the detector array. This source is scanned in time across the spectrometer slit, and the motion of the light source is converted into spatial along-track centroid and FWHM values (Fig. 16).

#### Uniformity

##### Spectral Uniformity and SUVs

The SRF, CRF, and ARF for the MISE instrument all exhibit small-scale differences in their centroid positions across the field, particularly in the visible wavelength portion of the detector. We call these small oscillations Spectrometer Uniformity Variations (SUVs). The SUVs appear to originate from the Dyson spectrometer construction and have also appeared in the EMIT imaging spectrometer, although at a smaller scale than in MISE. However, even with these spectrometer variations, the maximum spatial and spectral nonuniformity measurements are only slightly above the 10% pixel uniformity requirement, at 10.1% and 12.9%, respectively.

For example, the spectral uniformity is determined from the laser-sphere measurement used for the spectral calibration. The centroids for each laser line are fit to a curve in the spatial dimension, giving the amount of spectrometer “smile” or “frown” across the detector. The laser lines exhibit different smile and frown characteristics across the detector array due to the SUVs, as seen in Fig. 17. The reported spectral uniformity value is the maximum peak-to-peak nonuniformity of 12.9% of a pixel, which occurs at a wavelength around 1.750 μm. At the mid-wave infrared end of the spectral range, however, the measured spectral nonuniformity is as low as 2% of a pixel across the field.

##### Bad Pixel Map

The bad pixel map identifies pixels that do not respond to external illumination in a predictable way. These pixels are due to natural variability in the manufacture of detector unit cells and will be masked out in the processed data. We determine the bad pixel map by evaluating data across light sources to find dead pixels (pixels that always read low values), hot pixels (pixels that always read high values), pixels with high noise, and pixels that are more than 10% away from nominal responsivity compared with the median focal plane array. Generally, only a small number of pixels in the 480 × 320 pixel array detector will be identified as bad pixels. Bad pixels can also change over time, and these changes can be identified dynamically in flight using statistical methods.

The processing algorithms for previous imaging spectrometers, including EMIT and AVIRIS-NG (Chapman et al. 2019), use flight data to infer the true value of bad pixel array elements, replacing them with statistically appropriate values. These pixels are flagged so that future scientific analysis can identify the bad pixel array elements that have been replaced. A similar algorithm could be implemented in the MISE processing chain to replace bad pixel array elements.

#### Radiometric Calibration

Radiometric calibration allows translation from the measured raw data numbers (DN) at the sensor to incident radiance, which is important for scientific analysis of the data collected by MISE. We characterized the system radiometry by measuring illumination sources with known radiance properties. There are four components to the radiometric calibration: radiometric dark measurement, spectral radiometric calibration coefficients (RCCs), flatfield, and detector linearity.

##### Dark Measurement

In order to properly capture the dark current and any incident light on the detector that does not originate from our calibrated radiometric source, it is important to capture a dark measurement for each calibration measurement. Ideally, this dark measurement would capture the signal present with no external illumination. This includes signal due to the detector dark current, a bias from the readout electronics, and any self-emission of the instrument optics. A good proxy for the dark measurement is obtained during calibration testing by pointing the scan mirror to a view of the FPA, which has been validated as the darkest available view angle in the MISE instrument. The appropriate dark measurement view angle for in-flight activities will similarly need to be validated during in-flight calibration. Masked FPA rows are additionally used in each illumination measurement to estimate residual changes in the dark value over time. Figure 18 below shows an example dark frame. The vertical axis is the wavelength dimension, and the horizontal axis is the spatial dimension. Column-wise structures along with other more diffuse broad-area features are visible, which are due to detector and read-out circuitry characteristics.

##### Radiometric Calibration Coefficients

The radiometric calibration coefficients (RCCs) are multiplicative factors at each wavelength channel that translate measured DN to spectral radiance incident on the MISE instrument. These coefficients are given in units of μW/(nm cm^2^ sr DN). We calculate the coefficients by measuring calibrated sources with known radiances at the flight integration time of 53.47 ms to fully capture flight-like detector electronic effects. Since the MISE instrument covers a wide wavelength range with a varying response across that range, it is difficult to use a single source to calibrate the entire detector. The radiometric calibration is therefore divided into two distinct wavelength regions: the short-wave infrared (SWIR) from 0.8 μm to 2.5 μm, and the mid-wave infrared (MWIR) from 2.5 μm to 5 μm.

The SWIR radiometric calibration uses external National Institute of Standards and Technology (NIST)-calibrated sources which are relayed through the optical ground support equipment (OGSE) in the thermal vacuum chamber used for instrument testing. Given testing equipment limitations and uncertainties, we are using two distinct systems to calibrate the SWIR region: a NIST-traceable spectralon panel illuminated by a NIST-traceable lamp and a large spectralon-coated integrating sphere illuminated by a bright lamp. While the NIST-calibrated panel and lamp test provides the most direct radiometric measurement, the source is too bright to be measured in the SWIR region without saturation at the flight integration time and instead takes place with a 1.715 ms integration time. We translate the radiometric calibration at the 1.715 ms integration time by measuring its relationship to the flight integration time (53.47 ms) with a different light source. In contrast, the large spectralon sphere source’s output can be controlled by a variable aperture, which makes it easier to tune to different output light levels. A transfer calibration approach provides the radiance of the variable aperture sphere source, using an Analytical Spectral Devices (ASD) FieldSpec Pro field spectrometer that alternately measures the radiance output from sphere source and the radiance from a known NIST-calibrated lamp and spectralon panel. We constructed a calibration by concatenating RCCs from multiple lamp intensities that provide bright but non-saturating illumination to different parts of the spectral range. However, there are difficult-to-quantify uncertainties in the transfer calibration process due to changes in the light output as the ASD is moved, ASD nonlinearities, dynamic range issues, and variations over time. For both sources, independent measurements of the OGSE enable direct calculation of the radiance at the telescope aperture. Both radiometric calibrations of the SWIR region will be available for comparison with in-flight calibration using the Sun to determine the final SWIR RCCs for use at Europa.

The MWIR radiometric calibration uses a black silicon blackbody target fabricated at the Jet Propulsion Laboratory (Yee et al. 2017). The blackbody target is a nearly ideal blackbody source in the 2.5 μm to 5 μm wavelength region. The target is mounted inside the testing thermal vacuum chamber, which allows it to directly illuminate the MISE telescope and avoids absorption by atmospheric water vapor in the 2.7-μm region. During testing, we measure the blackbody temperatures at multiple temperatures from 135 K to 373 K, which gives good signal across the MWIR and into the SWIR region, covering the 2.3 μm to 5 μm region. The overlap between the MWIR and SWIR RCCs allow us to verify that our radiometric solution is consistent across the instrument’s full wavelength range.

##### Flat-field

The flat-field of the instrument (Fig. 19) provides a single scalar close to unity for every detector pixel, used to scale the FPA output such that a uniform input illumination returns a uniform output. The flat-field accounts for slight variations in sensitivity across the detector, including effects from detector array variations, read-out circuitry, the order-sorting filters, and other optical sources. The instrument flat-field is measured with a bright light source through an integrating sphere scanned spatially across the FPA. The peak signal for each pixel is divided by the average spectrum at the center of the detector, giving the multiplicative flat-field. The flat-field effectively normalizes the pixel responsivity across the detector, ensuring that the radiometric calibration is consistent across detector elements.

##### Linearity

While there is a large portion of the detector output that exhibits a consistent radiometric response, we expect that at low signal levels, the detector output will be nonlinear due to known detector characteristics. This results in a radiometric response for low signal levels that is inconsistent with the radiometry at high signal levels. Since MISE measurements will span the dynamic range of the instrument, it is important to develop a linearity correction to ensure that the radiometry is consistent across signal levels. The linearity correction is applied to all measured DNs after dark subtraction and translates from the raw signal to an ideal linear signal. The ideal linear signal is then converted to radiometric values using the flat-field and RCCs, as described above.

The MISE detector meets the requirements of the Europa Clipper mission, but exhibits higher nonlinearity than previous imaging spectrometers, including EMIT. Multiple tests were used to quantify the detector nonlinearity, including measuring many temperatures with the MWIR blackbody target, an integration time sweep, and a novel dual source method that measures the derivative of the nonlinearity at different signal levels. The results from these methods will be discussed in detail in an additional pre-flight MISE calibration paper. Figure 19 shows the preliminary linearity correction curve for MISE, which translates the input raw DN to output ideal DN.

## Overview of the Observations by MISE

This section describes the operation plan, from collection of the different science and calibration data types out to data release types and plan. Details on data volumes and processing are in Sect. 5.

### Observation Types

An observation by MISE will consist of spectral data for each point on the surface within the observation footprint, and thus is referred to as a “cube” (Fig. 20). Throughout each flyby, there will be three altitude-delimited classes of cubes collected (Table 5). As noted in the table, the altitude ranges are set based on small differences in the planned operations for cube collection and science analysis focus.

**Table 5:** MISE Observation categories

	Altitude range	Resolution	Notional co-adding	Primary science focus
Global scale (which includes the joint scan)	1200-40,000 km	< 10,000 m/px	20×	Determine the global-scale composition and chemistry and identify units and large-scale variability, to identify exogenic compositional signatures and search for possible large-scale heterogeneity in the ocean.
Joint scan with Europa-UVS, E-THEMIS, and EIS	∼32,000 km			
Regional scale	125-1200 km	< 300 m/px	20×	Determine the regional-scale surface composition and chemistry, to understand the chemical pathways between the ocean and surface and implications for the habitability of the ocean.
Local scale	Below 125 km	< 50 m/px	7×	Determine composition of individual landforms, to investigate how composition influences the formation and evolution of landforms.

The global scale observations start when the entire Europa disk fits into the field of view, at 40,000 km altitude, and are made during both inbound and outbound portions of the flyby (Fig. 21). Global-scale observations collected from lower altitudes contribute towards generation of a global composition map with spatial resolution <10 km/px. These measurements will map absorption features due to hydrated salts, organics, water ice in various phases, and radiolytic products (supporting mission objectives for identify and map endogenic/exogenic materials, determine the age of the surface, and understand ocean heterogeneity).

The local-scale observations consist of cubes obtained near closest approach to the target body. These observations will resolve smaller scale landforms. The altitude upper bound for these cubes is 125 km as that is the altitude where the potential orbit-determination error and spacecraft ground speed may be high enough to induce smear, and thus raw data will be downlinked, rather than in the form of cubes that have been processed and compressed onboard the spacecraft.

The regional-scale observations consist of cubes at altitudes between the global scale and the local scale. These observations will resolve larger scale surface features and map absorption features due to hydrated salts, organics, water ice in various phases, and radiolytic products (supporting mission objectives for pathways, habitability, and current activity). The altitude upper bound for these cubes is 1200 km as that is the altitude where scan mirror usage is expected to switch from scanning across the surface to compensation for spacecraft motion.

Dayside cubes are limited to observations of surface areas with local solar times of 9 am–3 pm local solar time (LST), and these are the focus for compositional investigations and coverage estimates (see Sect. 4.3.1). Nightside cubes, collected with local solar times of 6 pm–6 am LST from all altitudes, will contribute to searches for surface activity, specifically thermal anomalies. Additional observations acquired during 6–9 am and 3–6 pm LST could help clarify the light scattering properties of the surface.

### Typical Encounter Scenario and Timeline

During each Europa encounter, the MISE team plans to follow a general observation and operations sequence, which is described well by the notional encounter scenario illustrated in Fig. 21, which also defines the various subphases of an encounter. The exact timing and number of activities will be tailored to the science needs of a given encounter, but in general all activities will be completed in a similar order (also described in Sect. 5.2).

Starting with the end of encounter E_n_ and preparations for encounter E_n+1_: Since MISE has its own onboard memory with enough capacity to store 1–2 encounter’s worth of raw and processed data, the plan is to uplink and execute an onboard memory deletion command prior to the upcoming flyby after confirming that the instrument and processing operated as expected. Thus, approximately four days before the end of the encounter E_n_ (equivalently, six days before closest approach on encounter E_n+1_), the MISE science and operations team will have analyzed at least one processed image cube from that encounter to verify successful onboard processing. If an issue is detected, the data collected by MISE on that encounter can be reprocessed within the MISE memory. All processed cubes will be sent to the spacecraft memory (i.e., Bulk Data Storage (BDS)) to be stored until their downlink, but the raw data will be lost after the delete command is sent for the MISE memory. In addition, following the completion of the E_n_ encounter’s uplink planning process, the MISE team will generate and validate a preliminary command load for encounter E_n+1_, based on the current orbit determination predicts, which would be uplinked along with the onboard deletion command load.

Following the last orbit correction maneuver (three days before closest approach and one day before the start of encounter E_n+1_), the final orbit determination predicts are received by the MISE team. At ∼2 days before closest approach, the MISE team generates and validates an updated command load for the upcoming flyby. Prior to uplink, MISE is powered on via the spacecraft and the updated command load is uplinked for execution. The MISE cooling mode, which will cool the detector and spectrometer to operational temperatures, begins ∼40 hours before the first observations by MISE would be collected.

MISE Europa flyby data acquisition (+/− 40,000 km altitude) occurs on an operational continuum, but the individual data takes are categorized by the target range at the time of acquisition (see Table 5). The nominal MISE flyby data acquisition plan (also called the reference encounter scenario) consists of about six global-scale observations (including the joint scans with Europa-UVS (Retherford et al. 2024 this collection), E-THEMIS (Christensen et al. 2024, this collection), and EIS (Turtle et al. 2024 this collection), where the spacecraft attitude is used to acquire global scans of Europa from ∼32,000 km altitude), about three regional-scale observations, and one local-scale observation acquired at closest approach. The acquired ∼10 cubes are bracketed and/or interleaved by dark observations acquired through either a deep space observation or with the mirror in the closed position; further details of this cube collection are outlined in Sect. 5.2.

After the departure phase (∼two days after closest approach), MISE onboard data processing commences. The total time to process the data and transfer to the spacecraft for downlink is ∼24 hours. The data collected by MISE is downlinked periodically during the playback phase of an orbit and is available for ingestion by both the Europa Clipper Mission Science Data System and the MISE Science Data System ∼24 hours after receipt on the ground.

### Observations by MISE During the Jupiter Tour

Before launch, notional planning schedules (described in Sect. 4.2) were used to establish that sufficient MISE observations could be collected during the baseline tour to address key science objectives of the Europa Clipper mission. That analysis is shared here as a representation of the science data that MISE will collect, but MISE observation planning has a lot of flexibility that will be utilized in flight. In particular, the observations acquired by MISE during flight can be varied in length (number of lines), co-adds, and timing to enable careful focus on geological regions and features of interest as well as to build up an understanding of cumulative radiation effects and ocean and ice shell physical and chemical processes with global mapping of the surface spectra.

#### Europa Dayside Surface Coverage

**Fig. 3:**
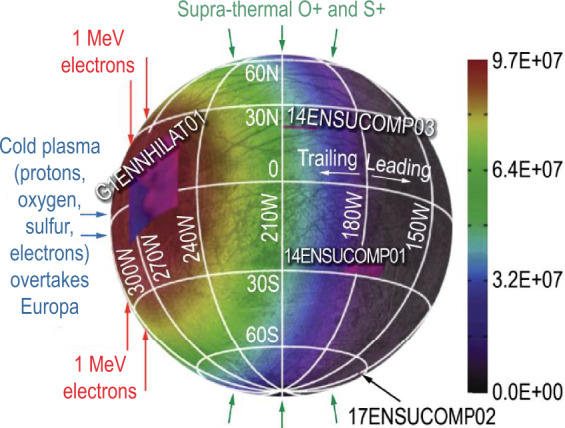
Simplified view from Dalton et al. (2013) of the predicted sulfur bombardment pattern (the color bar is sulfur ions per cm^2^/s) into Europa’s surface, using a model by Cassidy et al. (2013). Also drawn in this figure are other agents relevant to surface weathering. As shown, the leading and trailing hemispheres will experience substantially different fluxes and there will be latitudinal controls on the amount of processing of the surface. G1ENNHILAT01, 14ENSUCOMP03, 14ENSUCOMP01 and 17ENSUCOMP02 refer to Galileo NIMS observations

MISE will collect spectra of at least 60% of the Europan globe at 10 km/pixel or better (Fig. 22), and at least 0.3% (or ≥92,000 square km) at 300 m/pixel (Fig. 23). Special focus is paid to the centers of the leading and trailing hemispheres as well as the high-latitudes to address radiation investigations: the former so as to examine end-member areas with respect to identifying the effects of radiation swept onto Europa as it transits around Jupiter, and the latter so as to allow for detection of potentially less-degraded organic compounds due to the expected latitude-controls on irradiation-driven processing of the surface (Fig. 3). Additionally, the high-latitude observations will provide an assessment of volatiles that may not be stable in equatorial regions, including identification of potential cold-trapping regions near the poles.

**Fig. 4:**
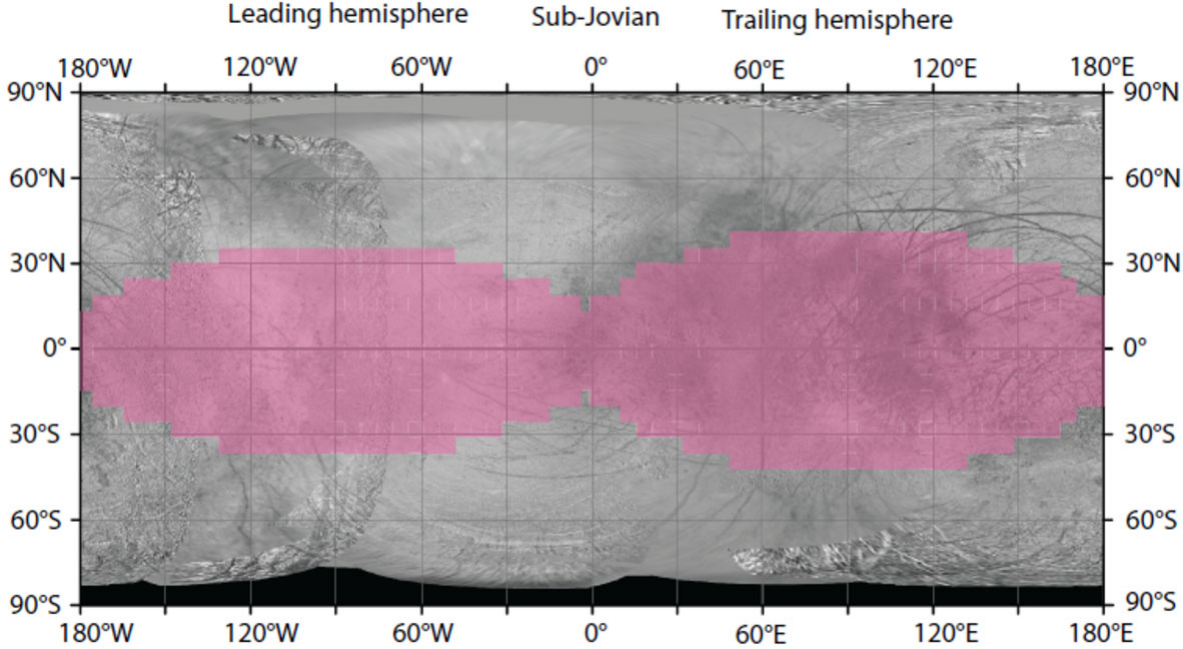
A map highlighting the main regions (in pink) on Europa’s surface where deep processing by energetic electrons takes place, one type of moon–magnetosphere interaction on Europa. 20 MeV (and higher) electrons move retrograde to Europa’s orbital motion and preferentially hit the leading hemisphere when their drift paths guide them over the moon. Figure from Nordheim et al. (2018)

**Fig. 5:**
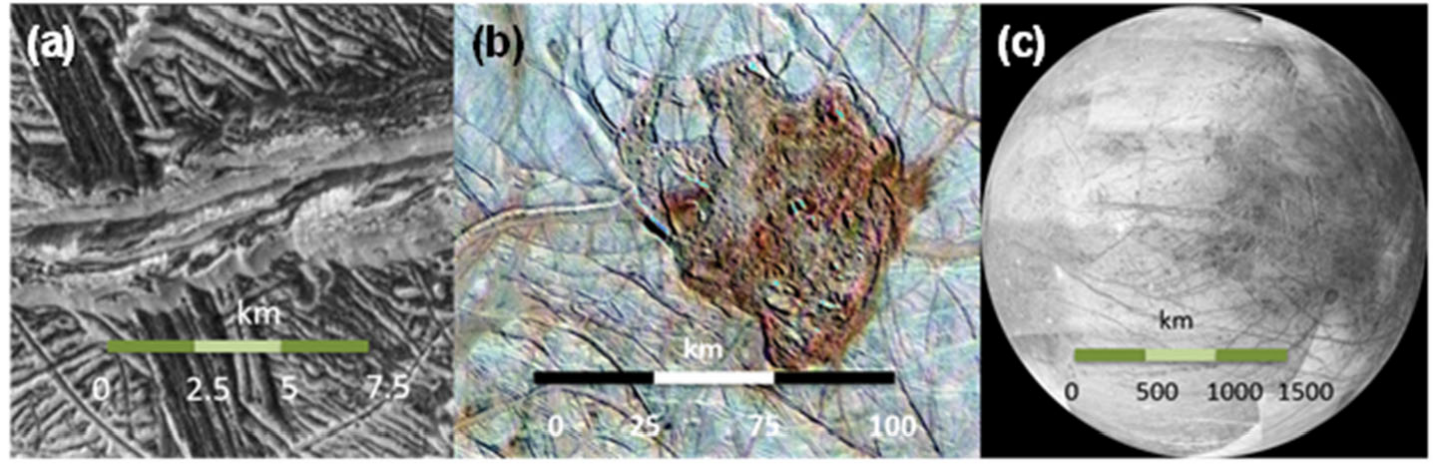
Galileo Solid State Imager (SSI) images of Europa at (a) 25 m/pixel, (b) 300 m/pixel, and (c) 7 km/pixel, which correspond roughly to MISE local, regional, and global spatial sampling performance. Note that albedo variations associated with different ice and non-ice components (salts, organics) are seen at all scales and reflect different processes that can be measured by MISE

**Fig. 6:**
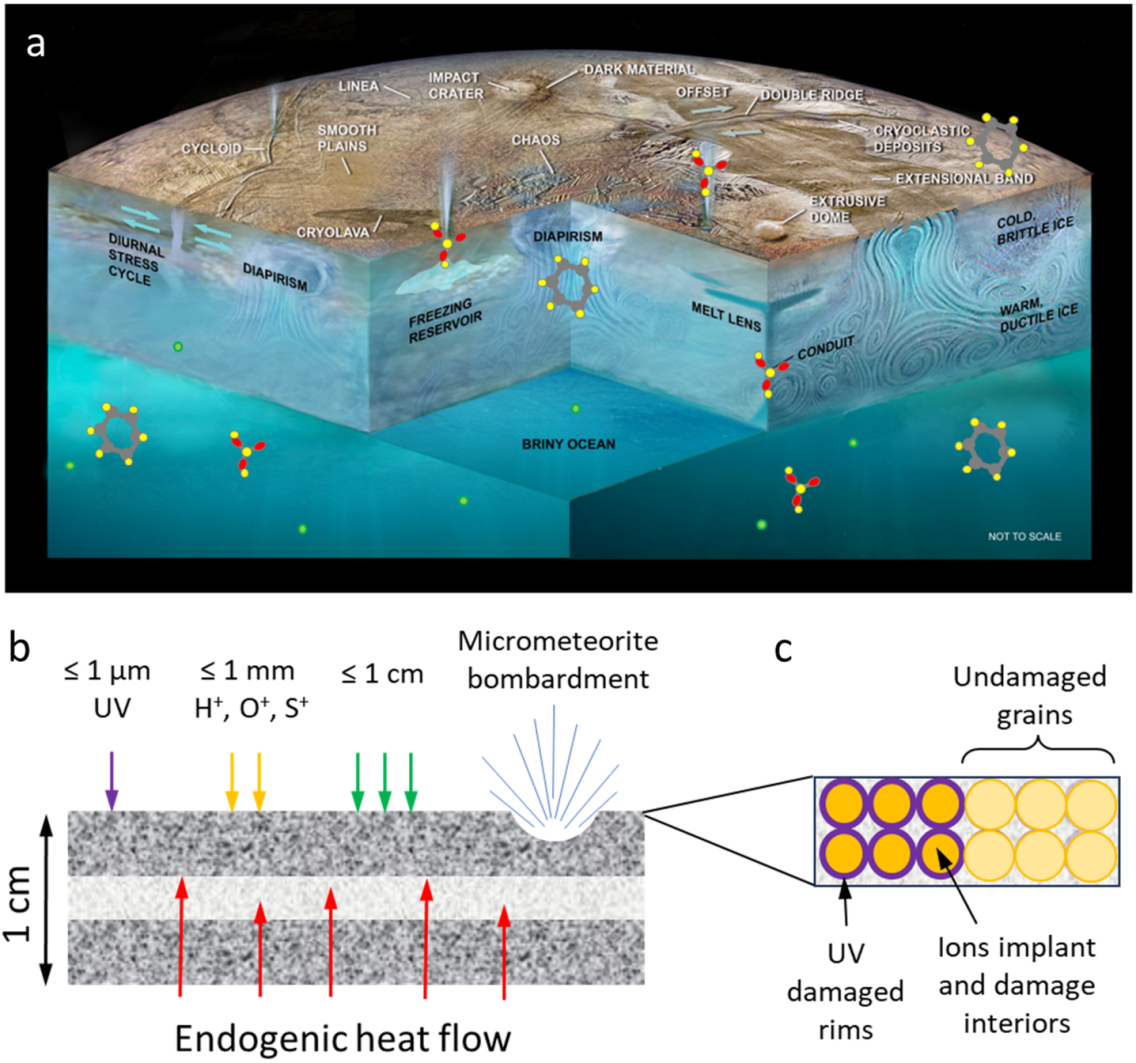
Many endogenic and exogenic processes detectable by MISE have the capacity to alter Europa’s surface chemistry and structure. (a) Sketch of macroscale geological and tectonic processes (described in detail in Daubar et al. 2024, this collection) that can transport organics and other molecules to the surface. On a microscale (b), radiation (colored downward arrows), particle bombardment, and endogenic heat flow (red upward arrows) can alter ice structure and chemistry through damage of exposed ice grains (c), changing their observed spectra

**Fig. 7:**
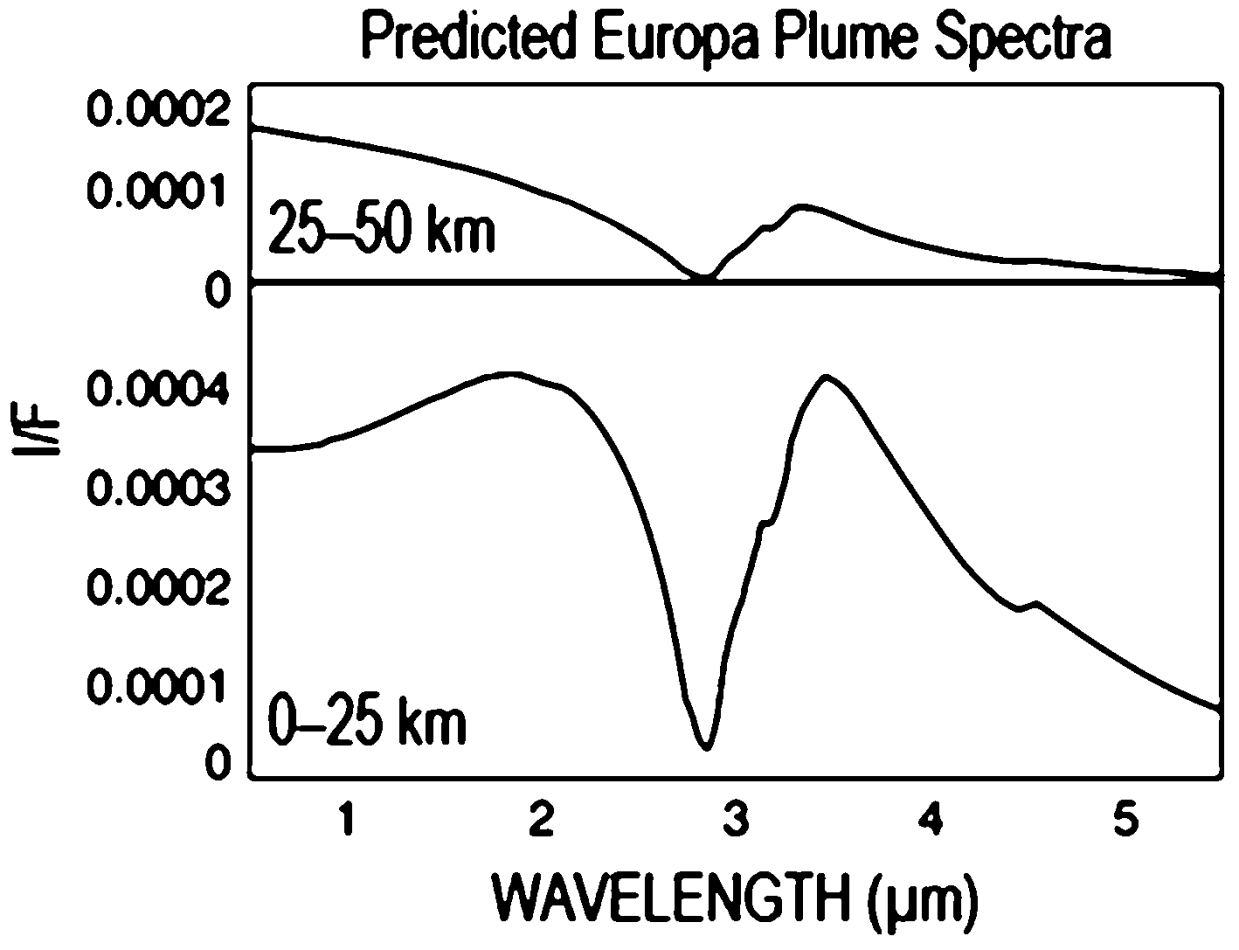
Two theoretical MISE spectra of a Europan plume, computed assuming that the plume particles are composed of pure water ice and that the particles’ size and launch velocity distributions are similar to those of Enceladus’ plume. The spectrum of the plume’s upper reaches is bluer than that of the plume’s base because larger particles are launched at lower speeds (Schmidt et al. 2008; Postberg et al. 2011)

**Fig. 8:**
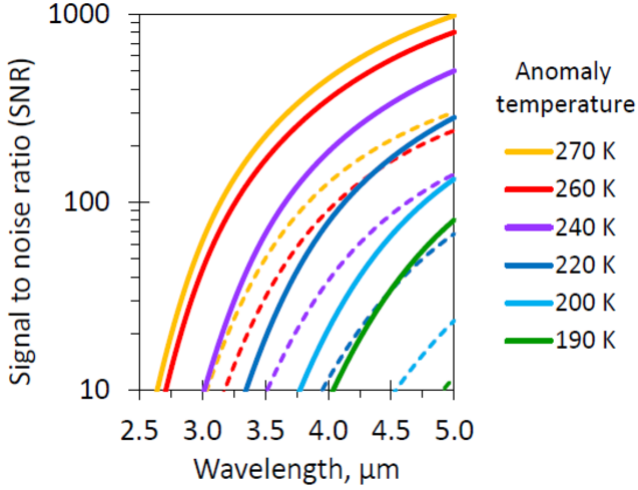
Detection of thermal anomalies by MISE. The solid lines represent a pixel-filling thermal anomaly. The dashed lines represent a 10% pixel fill fraction. The acceptable SNR is 10. Co-adding of bands further increases instrument sensitivity

**Fig. 9:**
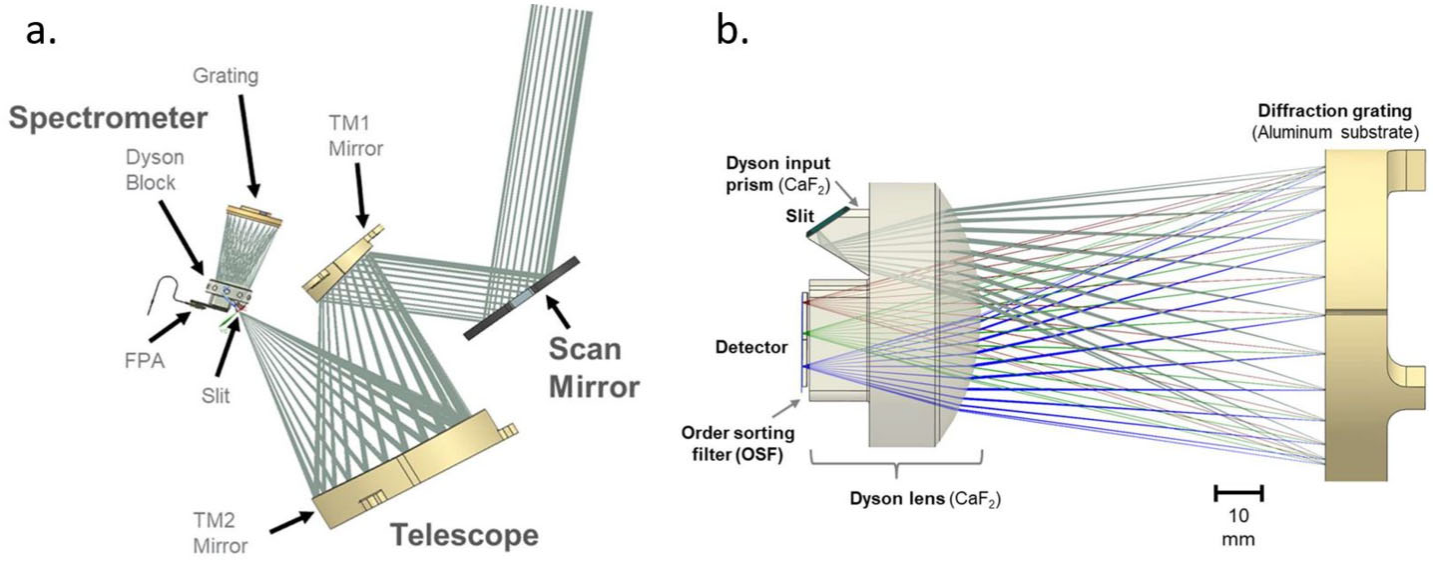
The MISE optical design (Bender et al. 2019). Figure 9a shows the MISE optical ray trace. The enlargement in Fig. 9b shows the MISE Dyson spectrometer ray trace in detail

**Fig. 10:**
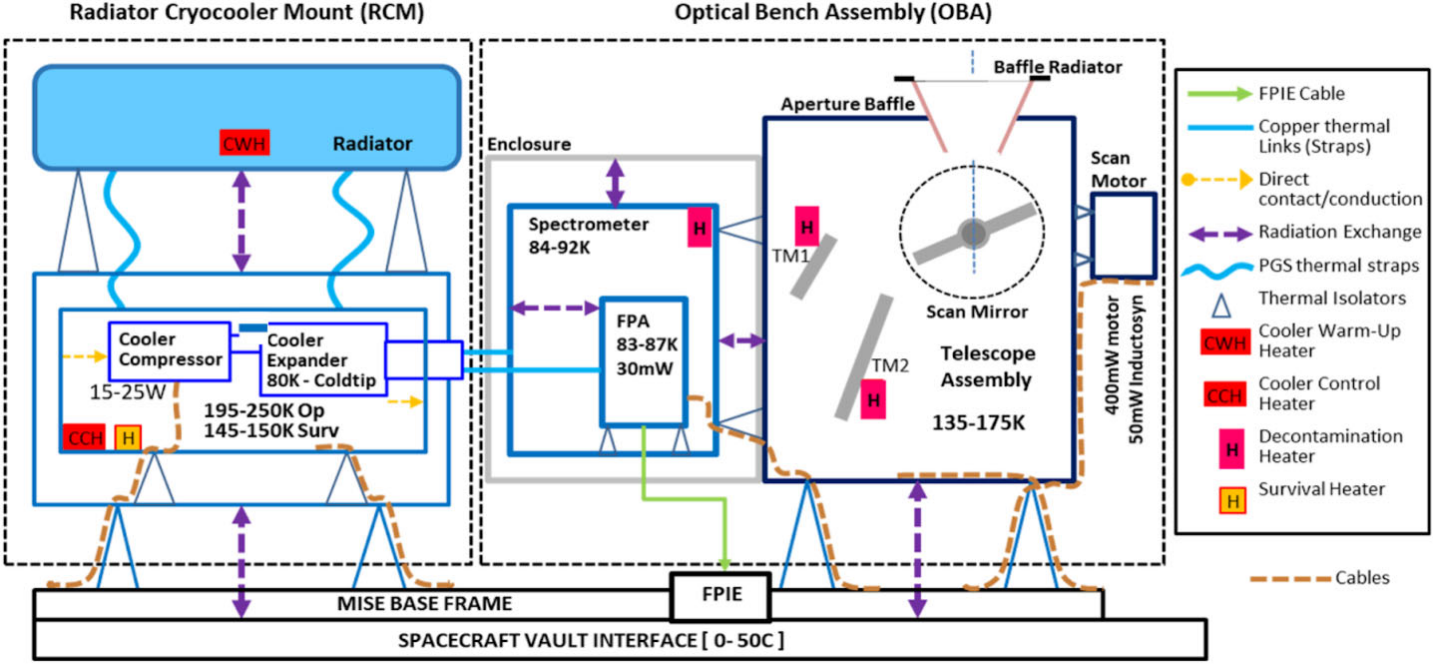
A schematic of the MISE thermal architecture with thermal control zones, temperature ranges, and heat loads

**Fig. 11:**
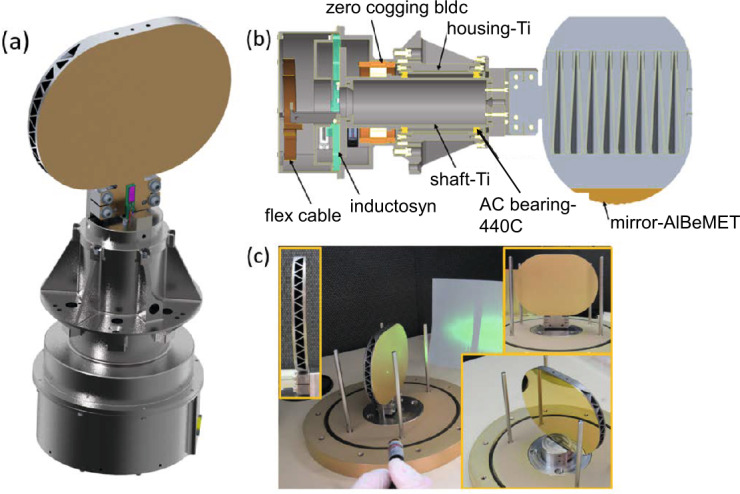
The scan system: (a) a computer-aided design (CAD) model of the scanner, (b) a cross-section view to highlight the mechanical approach of the cantilever design and lightweight AlBeMET mirror; (c) photos of MISE double-sided mirror in its shipping container; large image shows diffuse reflectance of a green laser at approximate operational calibration angles. Acronyms in the labels in (b) are explained in the text

**Fig. 12:**
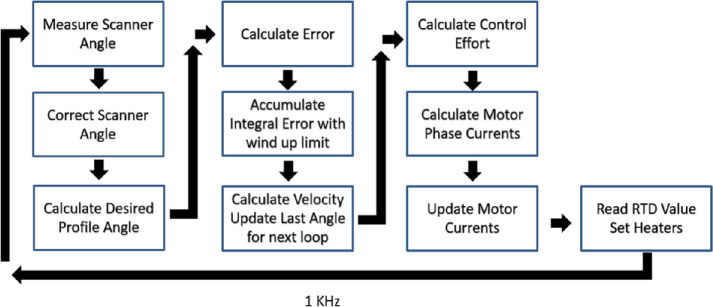
On-board processing steps for the MISE scanning control system

**Fig. 13:**
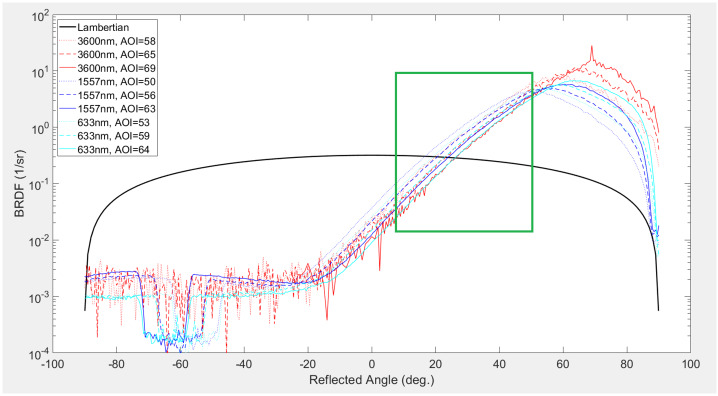
BRDFs of diffuse surface at 70° incidence angle; approximate operational range is depicted by the green box. AOI is the angle of incidence in degrees

**Fig. 14:**
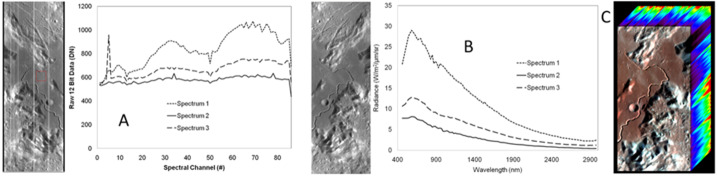
Example calibration of spectral observations based on laboratory-derived parameters, shown via the M3 imaging spectrometer at the Apollo 15 landing site on Earth’s moon (from Green et al. 2011) – representative of what will be done with MISE observations at Europa. (A) Raw signal in digital numbers (DN) per spectral channel. (B) Spectrally, spatially, and radiometrically calibrated spectra in units of spectral radiance. The calibration enables quantitative analysis of the measured spectra, which is crucial for scientific applications. (C) Fully calibrated M3 image cube delivered for science analysis

**Fig. 15:**
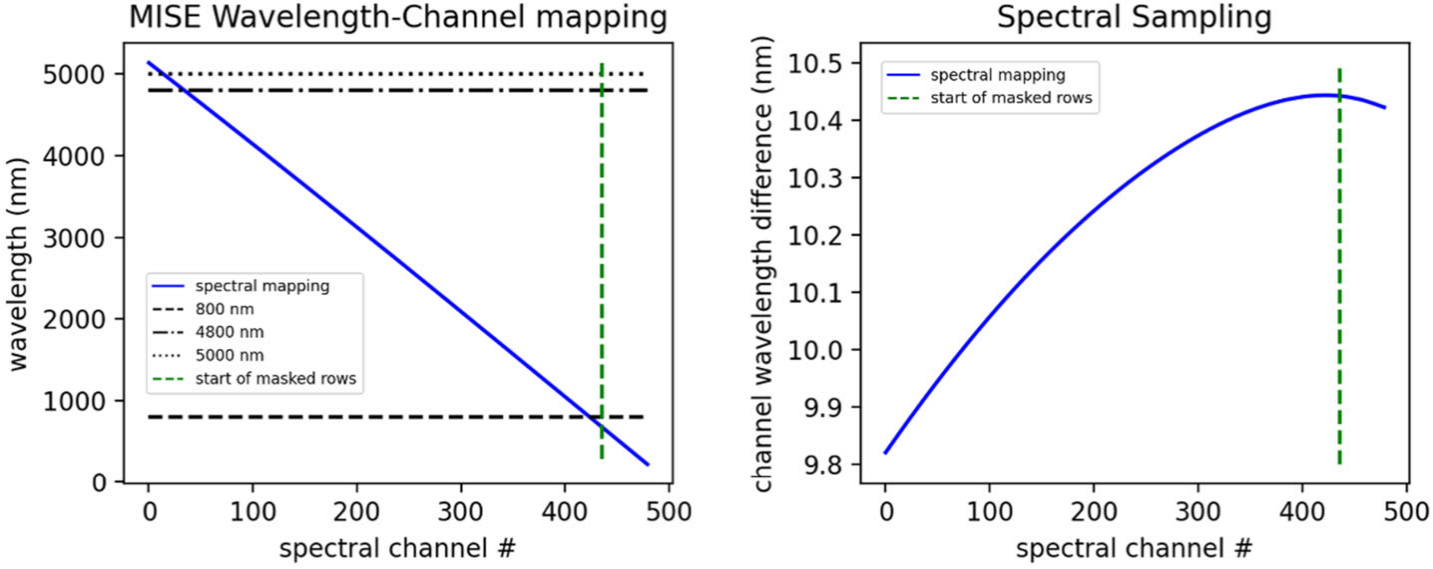
Wavelength calibration and spectral sampling curve, with masked rows (beyond row 435) and spectral requirements boundaries (800 nm and 5000 nm) indicated

**Fig. 16:**
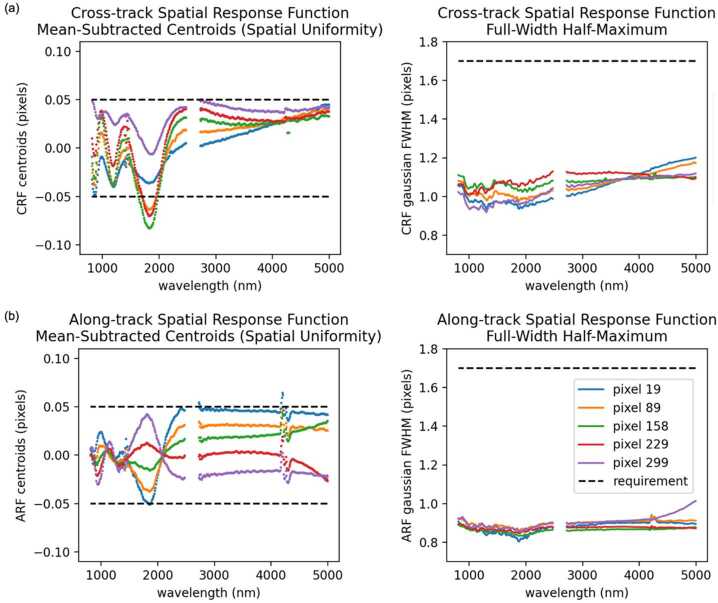
Spatial response function centroid errors and Gaussian FWHM. (a) Cross-Track Response Function (CRF) and (b) Along-Track Response Function (ARF) centroid errors both exhibit Spectrometer Uniformity Variations (SUVs). The vertical axis represents the magnitude of deviation from perfect uniformity. The bonded gap between the order sorting filter (OSF) and linear variable filter (LVF) at 2600 nm has low signal, and thus data in this region is not displayed

**Fig. 17:**
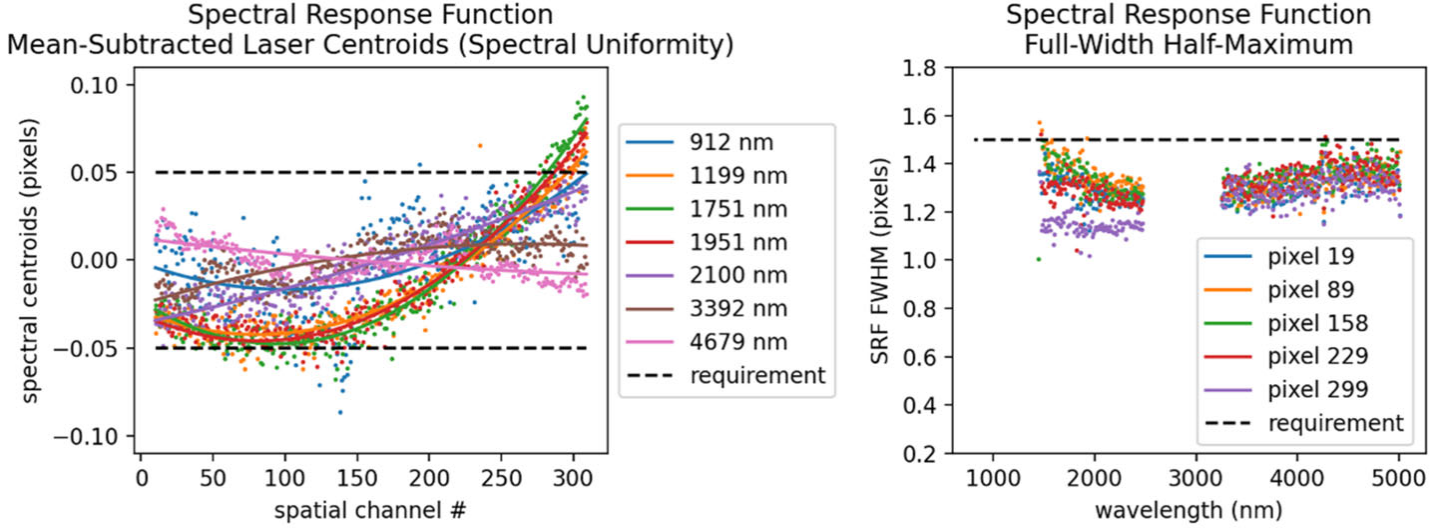
Spectral response function centroid errors for select laser wavelengths and spectral Gaussian FWHM across the spatial field. The vertical axis represents the magnitude of deviation from perfect uniformity. Spectral nonuniformity is low in the wavelengths above 2.1 μm, with low peak-to-peak centroid nonuniformity across spatial columns. Spectral nonuniformity is higher in the short wavelengths (below 2.1 μm), another example of Spectrometer Uniformity Variations (SUVs)

**Fig. 18:**
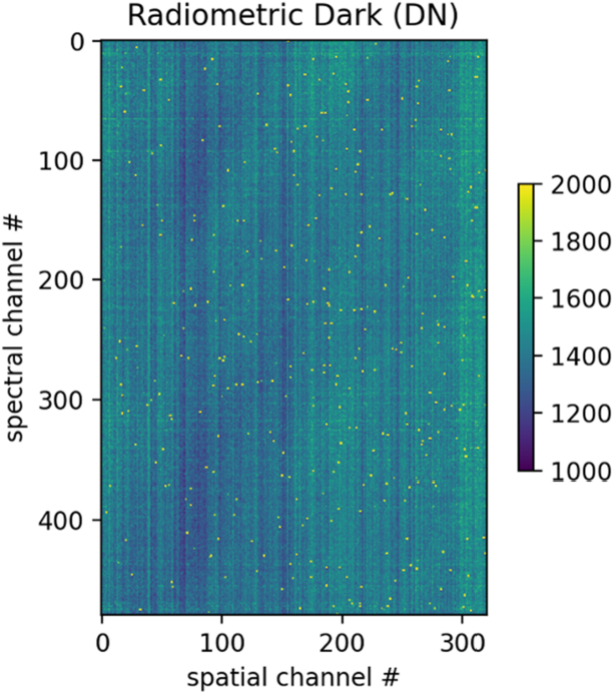
An example MISE dark frame

**Fig. 19:**
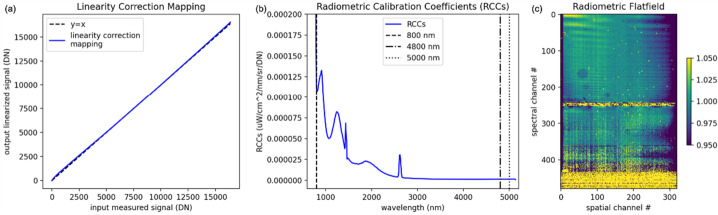
Initial a) linearity correction mapping, b) radiometric calibration coefficients, and c) radiometric flatfield results from recent instrument testing

**Fig. 20:**
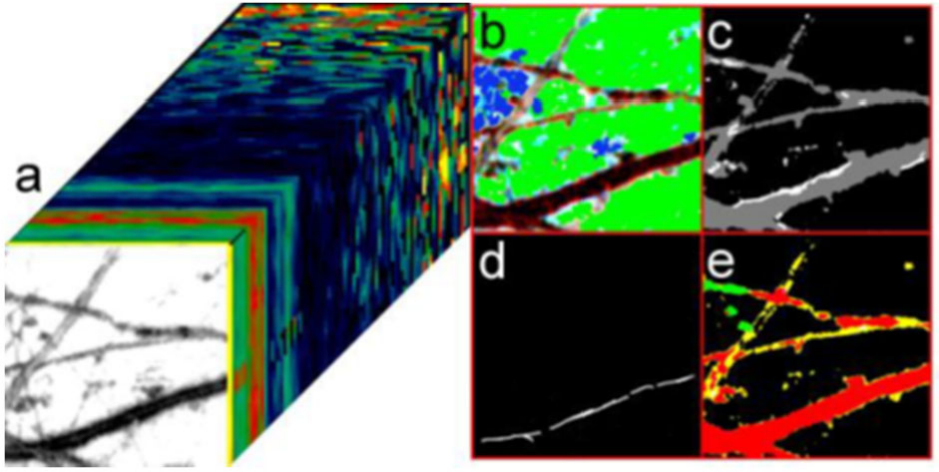
This synthetic Europa image cube illustrates how MISE cubes contain several types of compositional information that will aid the assessment of habitability. (a) 1 μm albedo map of the surface with the full spectrum and compositional information at each pixel extending backwards. (b) Map of ice phases: red = acid hydrate, green = crystalline ice, blue = amorphous ice. (c) Distribution of epsomite, a salt, (d) Map of thermal anomalies, and (e) Map of epsomite (red) and two organics: benzene (green) and octane (blue). Yellow areas have both epsomite and benzene. MISE shows this area contains multiple spectral tracers of habitability: salts, temperatures indicative of recent activity, and organics associated with bands, a landform hypothesized to reflect transport of material from within the ice shell

**Fig. 21:**
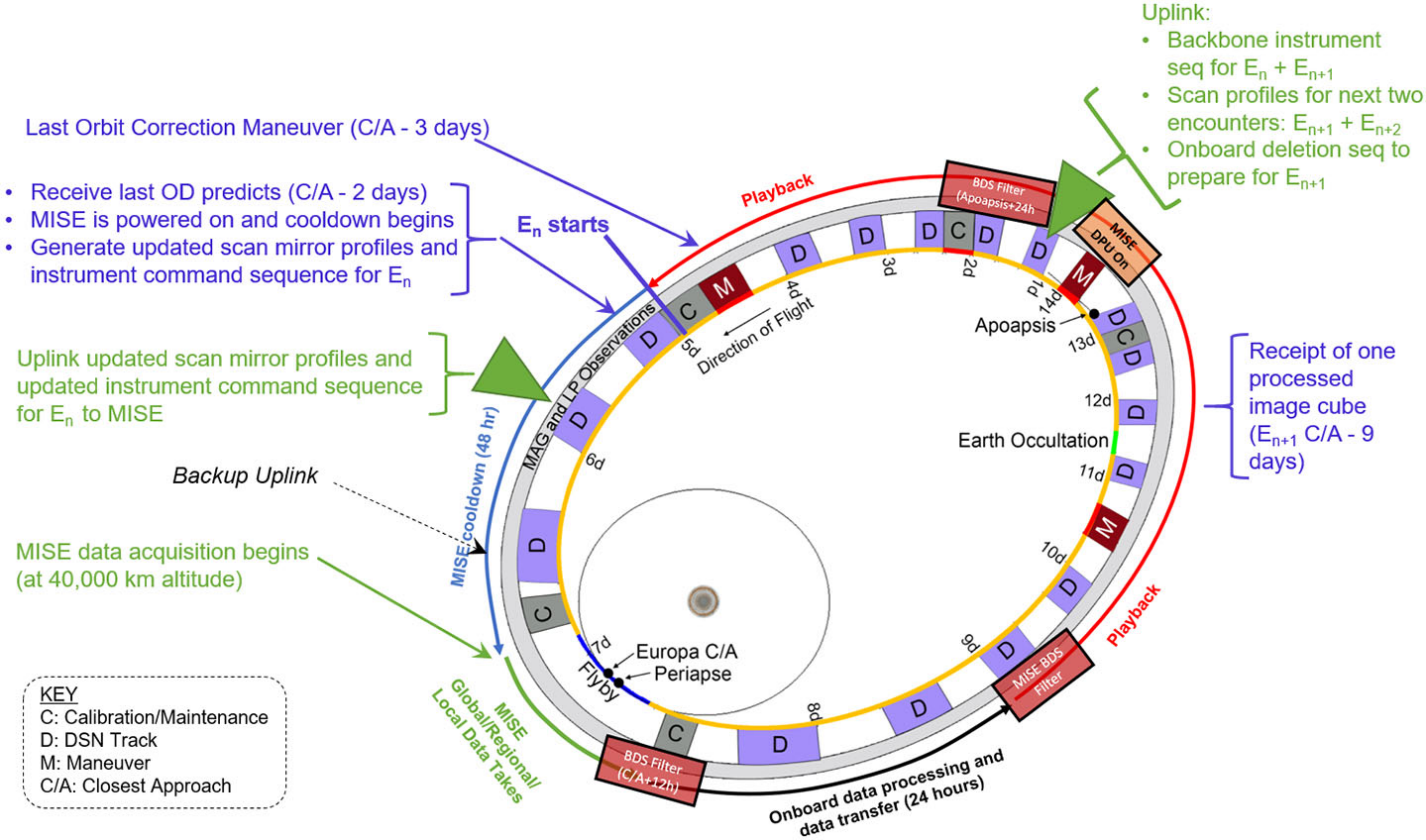
A schematic representation of the MISE activities that will occur during each Europa flyby. An encounter (E_n_) is defined as starting 2 days before closest approach (C/A) h (i.e., at about the five-day (5d) marker, where days are counted from apoapsis). In addition to the observation acquisition strategy explained in 4.1 (and during the green curve) and data validation/downlink plan explained in 4.2 (see receipt of one processed cube on right and green triangle near apoapsis), key events include (1) around the start of the E_n_, the spacecraft team will generate the last orbital determination (OD) predicts and MISE then generate and uplink their scan mirror profiles and command sequence for E_n_. The MISE cooldown will also start, bringing the instrument to its operational temperatures for data acquisition. The spacecraft’s Bulk Data Storage BDS filters (red boxes) are part of the spacecraft sorting of data for downlink; as MISE collects its data on-instrument for processing before sending to the spacecraft (explained in Sect. 5.1), a BDS filter will run about two days after C/A so that all MISE data from E_n_ can be processed and readied for downlink

**Fig. 22:**
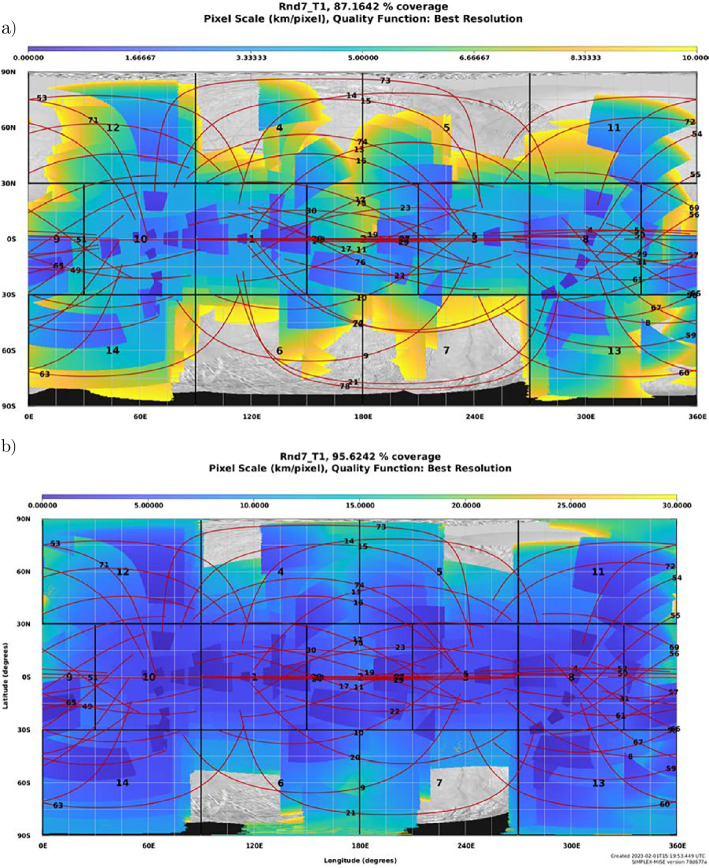
The global-scale coverage achieved with the notional observation plan for tour Rnd7_T1; this map is representative of the observations that MISE would collect. During flight, targeting may be a little different as that would include consideration of interesting geologic units or features, maximizing unique coverage with higher resolution observations, and other science priorities of the Europa Clipper science team. (a) This map shows the coverage that would be used for generation of a near-global map better than 10 km/pixel in resolution; in this simulation the achieved coverage is 87.2%. Also shown are the regional-scale images (small, dark blue boxes), which could also be integrated into this map. (b) This map shows the resolutions that would be achieved at better than 30 km/pixel in resolution (i.e., the areas 10–30 km/pixel would be imaged with the observations shown in (a) and useable by the science team, but not counted towards the Europa Clipper Level-1 science requirements); in this simulation the achieved coverage is 95.6%. These lower-resolution observations are of science value; for example, even lower-resolution imaging of the polar regions is of interest for a search for cold-trapped volatiles. These two maps were generated with CADMUS using SIMPLEX with a simple algorithm for scheduling, as outlined in Sect. 4.2

**Fig. 23:**
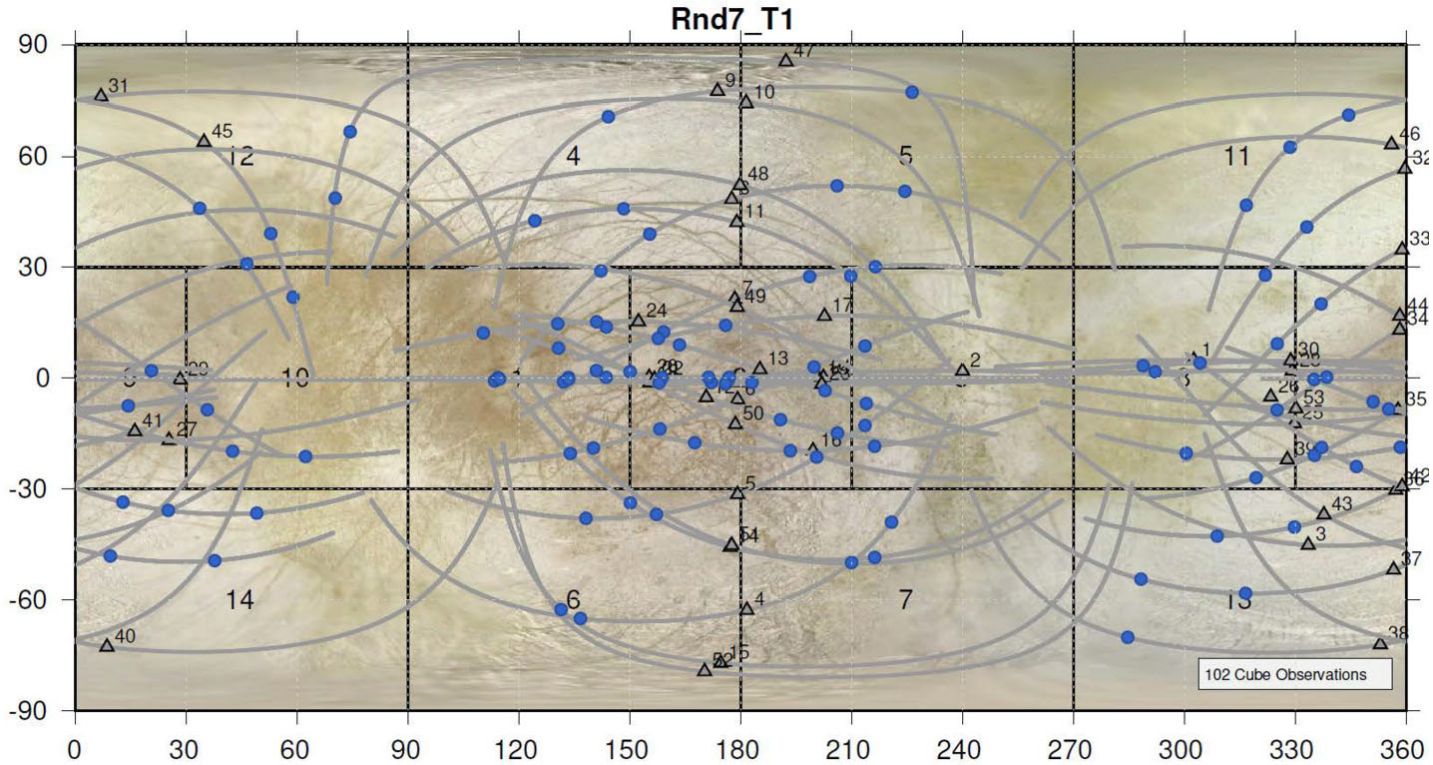
The regional-scale coverage achieved with the notional observation plan for tour Rnd7_T1; this map is representative of the observations that MISE would collect. Coverage shown is 0.61%. During flight, targeting may be a little different as there would be consideration of interesting geologic units or features, maximizing unique coverage with higher resolution observations, and other science priorities of the Europa Clipper science team. The map was generated with CADMUS using SIMPLEX with a simple algorithm for scheduling, as outlined in Sect. 4.2

As described in Sects. 4.1 and 4.2, some of the MISE global-scale cubes will be collected during the joint scan, which occurs just within 40,000 km altitude and involves the spacecraft scanning across Europa for E-THEMIS (Christensen et al. 2024, this collection), Europa UVS (Retherford et al. 2024, this collection), and EIS (Turtle et al. 2024 this collection). During these scans, the MISE scan profile will compensate for the spacecraft motion.

**Fig. 24:**
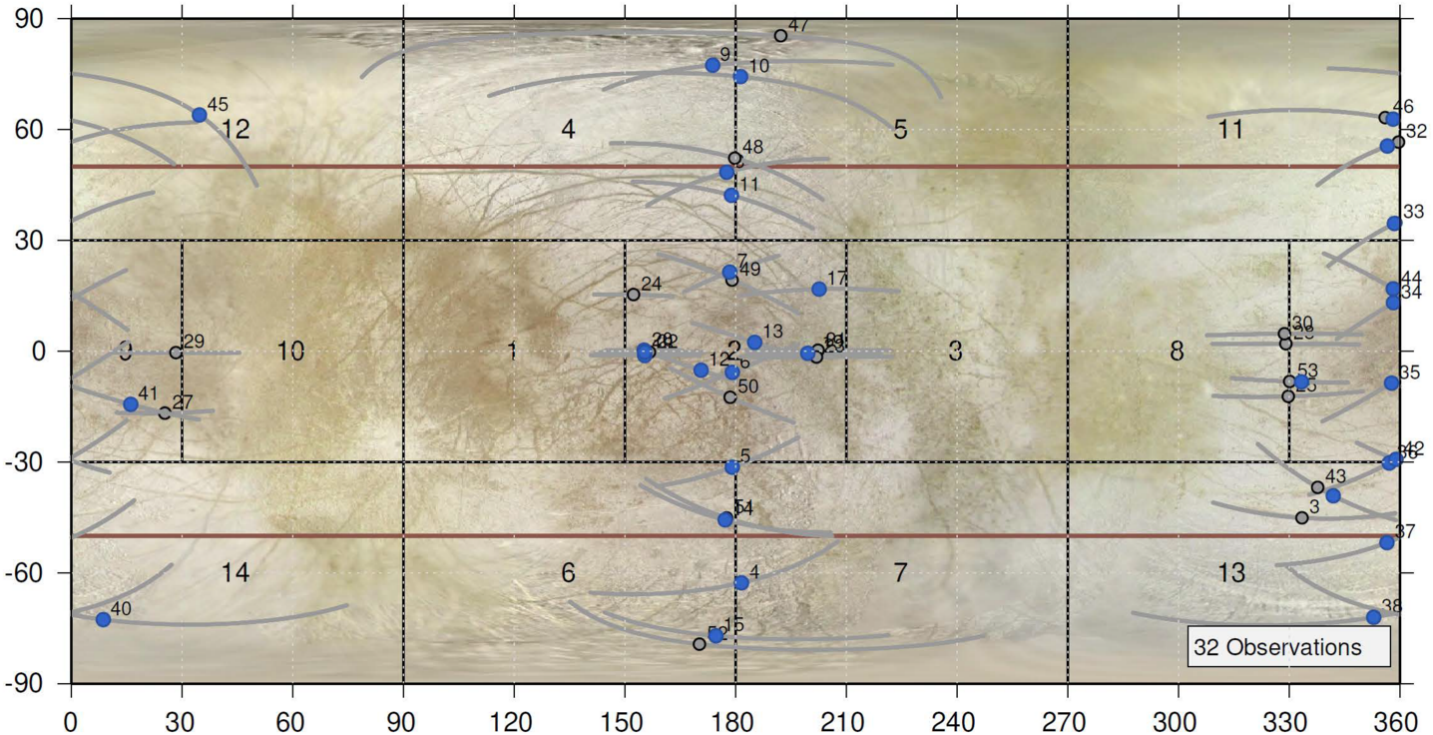
The local-scale coverage achieved with the notional observation plan for tour Rnd7_T1; this map is representative of the observations that MISE would collect. During flight, targeting may be a little different as there would be consideration of interesting geologic units or features, maximizing unique coverage with higher resolution observations, and other science priorities of the Europa Clipper science team. The map was generated with CADMUS using SIMPLEX with a simple algorithm for scheduling, as outlined in Sect. 4.2

Local-scale cubes are collected whenever the altitude and local solar time (LST) requirements are met; in this tour, 32 are planned, including three per hemisphere that are poleward of 70° (Fig. 24). Due to the flyby geometry, all local-scale cubes are within ∼30° of 180° E and 360° E longitude.

#### Europa Nightside Observations

**Fig. 25:**
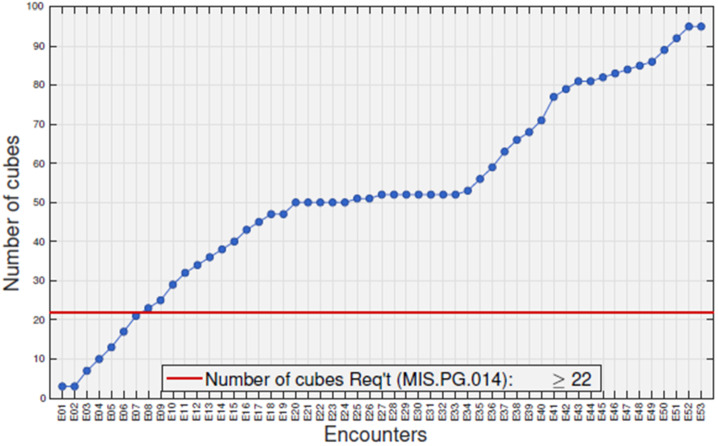
The number of nightside cubes collected via the notional observation plan for tour Rnd7_T1; this map is representative of the observations that MISE would collect. During flight, targeting may be a little different as there would be consideration of interesting geologic units or features, maximizing unique coverage with higher resolution observations, and other science priorities of the Europa Clipper science team. The map was generated with CADMUS using SIMPLEX with a simple algorithm for scheduling, as outlined in Sect. 4.2

Night-side observations (i.e., cubes collected from 6 pm to 6 am LST) will be used to detect surface thermal anomalies (including, potentially, ongoing resurfacing activity) and to map thermal variations globally (which then will be correlated with regions and landforms). At least 22 nightside cubes are to be collected from altitudes below 40,000 km, and notional observation simulations currently have nearly 100 cubes collected with broad spatial distribution (Fig. 25). The science value of these observations is described in more detail in Sect. 2.3.3, and targeting may be influenced by E-THEMIS detection of hot spots (Christensen et al. 2024, this collection) or other indications of recent geologic activity and potential small-scale heating of the Europan ice shell.

### In-Flight Calibration Activities

In-flight MISE calibrations will encompass spectral, spatial, radiometric, flat-field, and noise checks. Some of these calibration activities involve regular measurements before and after Europa observation cube acquisition, and others involve special observations of Jupiter, other Jovian moons, the Sun, and stars (Table 6). (Pre-flight calibrations were described in Sect. 3.8.) Each activity is collected at least once per listed mission phase, except for the dark calibrations which typically bracket all other MISE measurements (see Sect. 5.2). Since the dark calibrations are always to be collected before and after calibration events in flight, their data volume has already been added for each of those calibration activities. The data volumes listed show what would be sent to the spacecraft without compression, and details about data volume are in Sect. 5.

**Table 6:** Summary of MISE in-flight calibration measurements

Cal. Type	Objective	Source/Target	Mission Phase	Data Volume, per Activity
Spectral	• Wavelength (methane, CO, water vapor lines) • Uniformity (cross-track)	Jupiter	Transition to Europa Campaign 1 (TEC1)	15.6196 Gb
			Europa Campaign 1 (EC1)	
			Europa Campaign 2 (EC2)	
	Wavelength (SO_2_ Lines)	Io	TEC1	5.7024 Gb
			EC1	
			EC2	
Spatial	Uniformity (geometric; PSF)	Europa Limb	EC1	15.6196 Gb
			EC2	
		Star	Outer Cruise to Jupiter Approach	6.9420 Gb
			TEC1	
			EC1	
Radiometric	Intensity Calibration	Star	Outer Cruise to Jupiter Approach	25.5367 Gb
			TEC1	
			EC1	
	Intensity Calibration and Flatfield (freebie)	Sun	Outer Cruise to Jupiter Approach	1.4876 Gb
			TEC1	
			EC1	
			TEC2	
			EC2 (end)	
Flatfield	Full Optical Path	Callisto	TEC2	3.2231 Gb
Noise	Radiation (Characterization and algorithm performance)	Deep Space	TEC1	31.9829 Gb
Dark	Signal (DC)	Closed Mirror	All	0.7438 Gb per pair
		Deep Space (opportunistic)		

*Spectral calibrations* will include checks on wavelength accuracy, via methane, CO, water vapor, and SO_2_ lines—the last from observations of Io, and the others from observations of Jupiter. Concurrent with the Jupiter observation will be a uniformity (cross-track) check. At least three observations of Jupiter and three of Io will be collected; Jupiter observations will be collected from between 15 R_J_ and 25 R_J_, and the Io observations will be acquired when Io is within 25 R_J_ of the spacecraft (so that Io will fill at least eight pixels in the cube) and Jupiter is at least 15 R_J_ away. The target must be within the slit, 4.3° wide, (i.e., cross-track) and MISE will perform along-track scanning with the mirror. The first set of calibration observations of targets other than Europa will be collected within two weeks of the first Europa flyby, and an additional observation will be collected within the first two flybys during each Europa Campaign period (i.e., periods with specific operational characteristics: Outer Cruise to Jupiter Approach, Transition to Europa anti-Jovian hemisphere (TEC1), close viewing access of anti-Jovian hemisphere (EC1), Transition to Europa sub-Jovian hemisphere (TEC2), and close viewing access to sub-Jovian hemisphere (EC2); further details can be found in Pappalardo et al. 2024, this collection).

*Spatial calibrations* will be collected via observations of the Europa limb and standard stars, such as those listed in Stewart et al. (2015). The plan for the Europa limb observations is to fill the cross-track FOV with the disc of Europa and point the mirror so the limb falls on 3–4 locations of the MISE slit, while the spacecraft is ∼40,000 km from Europa (like the upper bound for the global-scale cubes, see Sect. 4.2). For the star observations, the spacecraft will be further than 15 R_J_ from Jupiter and the target will be within the 4.3°-wide slit with the spacecraft doing cross-track scanning to place the start in 3–4 locations (separated by approx. 1°) on the MISE slit, each lasting at least 2.5 min. The first star observations will be collected during (1) the outer cruise, no later than within one month before JOI and (2) no later than 2 weeks before first flyby. All other spatial calibration observations will be collected within the first two flybys during each Europa Campaign (Table 6).

*Intensity radiometric calibrations* involve observations of stars, such as those listed in Stewart et al. (2015), and the Sun, and are acquired when Jupiter is more than 15 R_J_ away. The solar observations will also yield flatfield calibration information. Solar observation will be collected through up to 10 pre-set MISE mirror positions (no spacecraft pointing changes needed). Star observations will include an along-track scan with the star within ±2° of the center in the cross-track direction. The first radiometric calibration observations are to be collected during (1) the outer cruise, no later than within one month before JOI and (2) no later than 2 weeks before first flyby. All other radiometric calibration observations will be collected within the first two flybys during each relevant Europa Campaign or Transition to Europa Campaign period (Table 6).

*A flat-field calibration*, using the full optical path, will be collected via observation of Callisto with phase angle <90° from <30,000 km altitude or with phase angle <20 from 30,000–60,000 km altitude from Callisto. Callisto will be within the 4.3°-wide slit and MISE will use the scan mirror to capture the target. This observation will be collected during the transition to Europa Campaign 2, within the two weeks before the first flyby in that campaign.

*Noise-focused calibration observations* aim to characterize the radiation around Europa and the performance of the onboard MISE data-processing algorithm (described in Sect. 5.2). These observations will be collected between 9 and 10 R_J_ from Jupiter (i.e., from about the Europa orbit distance), with the MISE boresight pointed towards dark space, no later than one month before the first flyby.

*Dark calibrations* will check for signal noise during cube collection and will primarily involve observations of the back of the scan mirror before and after each cube (or series of cubes if a few are scheduled in close timing, such as during regional-scale observations, see Sect. 4.1). These are relatively short calibration observations with a 1-minute data-acquisition duration each while all previously described calibration observations have four to ∼30-minutes data-acquisition durations.

One additional planned calibration activity is the check on instrument and scanning alignment, which will occur as each planned observation is compared to the resultant acquired observation, after adjusting for the post-flyby knowledge of the spacecraft position/pointing and MISE pointing.

### Quicklook and Derived Products

To enable quick team analysis and guide cross-instrument observation comparisons, in-flight instrument performance analysis, and planning for future observations, a few data products will be automatically processed and released to the Clipper science team within two days of downlink. These will include:

- Calibrated spectral radiances for each cube
- Calibrated surface reflectance data
- Nighttime thermal emission maps
- False color (RGB) images
- Band depth images for specific compositional absorptions
- Ice parameter maps

The false color images are planned to be in .png format, and all the mapped data are planned to be in a binary format with detached ASCII header. For all types, sensor-space and geolocated data will be generated. After the spectral characteristics of Europa are better understood, additional quicklook data products may be created.

Similar quicklook and related derived data products have been used on MRO’s CRISM investigation (Murchie et al. 2007). In particular, ice (and other compositional) parameter maps for Mars have been defined in Viviano et al. (2014).

### Data Release Plans (PDS, Public)

MISE Raw and Calibrated Data Products will be delivered to the Planetary Data System (PDS) Geosciences Node according to the Europa Clipper project data delivery policy. A key element of the data delivery policy is the need for a reasonable interval of time to generate and validate Standard Data Products before delivery of Archival Data Products to the PDS. Based on numerous past mission experiences (including the Galileo, Cassini, Mars Global Surveyor, Mars Odyssey, and Mars Reconnaissance Orbiter projects) and the nature of the Europa Clipper mission, as much as six months could be necessary to produce useful and accurate Calibrated Data Products from Europa Clipper instruments. Raw and Calibrated Data Products should be produced, validated, and delivered to the PDS as soon as is practical, with the six-month period considered to be a maximum. The six-month period will be measured from the time that all required data including directly relevant ancillary data (including reconstructed ephemeris and attitude information) are made available by the Europa Clipper project to the MISE team. MISE Derived Data Products, such as ice grain size maps, are expected to take longer to generate than Calibrated Data Products and will be archived after the end of the prime mission.

## Data Processing

### Science Data Acquisition and Downlink

MISE data acquisition consists of reading full-frame data [x, $\lambda $] from the focal plane at the operational (53.47 ms) frame rate. The nature of a given set of continuous frames (described as an image cube or a frame stack) depends on what is occupying the MISE FOV as the frame data are acquired. Science data are acquired when MISE is viewing Europa and using the scan mirror to control the IFOV advance along the surface. Europa science observations can be acquired throughout the encounter phase (within 40,000 km altitude, as described in Sect. 4.1). Calibration data, described in Sect. 4.4, are acquired using sources other than Europa (such as Jupiter and the other Galilean satellites, stars, the Sun) with the scan mirror being used to stare, scan, or provide the appropriate observation geometry in combination with the spacecraft attitude. Dark frame data are acquired either by off-pointing the mirror to view space or closing the mirror.

When taking science data, MISE oversamples the target using the scan mirror to precisely control the advance of the single-frame FOV across the surface. When distant from Europa, where the apparent motion of the body is small, the scan mirror actively moves the MISE FOV across the body (i.e., the global-scale cubes described in Sect. 4.1). At lower altitudes the apparent motion exceeds the appropriate angular rate and so the scan mirror performs image motion compensation (IMC) (i.e., the regional- and local-scale cubes described in Sect. 4.1). The scan motion profiles vary significantly with altitude, but in all science data acquisition scenarios the scan motion is smooth and continuous. At all altitudes the along-track oversampling allows for the mitigation of radiation noise and the recovery of the required SNR through the evaluation and aggregation of each set of oversampled frames into a single resultant frame. For the nominal 20× oversampling and 20:1 frame aggregation this represents a corresponding 20:1 ratio between the acquired and downlink (D/L) data volume (ignoring compression).

All frame data are collected with MISE in the operational Data Aquisition mode and stored directly (and redundantly) to the onboard instrument flash non-volatile memory (NVM). The radiation noise remediation/frame aggregation processing is performed by the MISE Data Processing Unit (DPU) in the operational Processing Mode. The aggregate frame and related data resulting from the frame data processing are written back to MISE flash alongside the acquired data. Any data in the instrument flash (acquired and processed frame data, bookkeeping products and related statistics) can be transferred to the Spacecraft Bulk Data Storage (BDS) in the operational Transfer Mode. The data transfer handling supports row-reordering and subframing (together enabling wavelength selection), binning, in-line lossless compression, and packetization. The instrument supports two data transfer rates: (1) a fast rate of 3.260 × 10^−3^ Gb/sec, which is essentially a direct transfer, that applies to calibrations and raw cubes, and (2) a slower rate of 9.1 × 10^−4^ Gb/sec that is used for image cubes and other data that has been handled, as listed above.

MISE data packets include header information that provides commanding traceability (MISE macro sequence / target ID), unique product identification (accountable product ID), and D/L priority (BDS bin ID), which collectively inform the spacecraft BDS of the data product handling pathway to D/L.

**Fig. 26:**
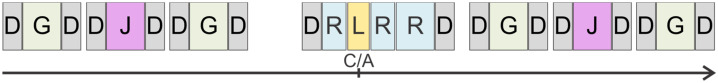
A schematic of the MISE reference Europa encounter data acquisition scenario. Each global-scale [G] and joint scan [J] cube (40,000–1200 km altitude) is bracketed by dark frame stacks [D]. Since observing time surrounding closest approach (C/A) is limited, dark frame stacks bracket rather than interleave with the regional [R] and local [L] observation set (<1200 km altitude)

As described in Sect. 4.1, the MISE instrument will collect cubes in different altitude ranges to address global-, regional-, and local-scale science. In the reference encounter scenario (Fig. 26), each of these observation types follow different data handling pathways and result in different data volumes and product rosters being sent to the spacecraft BDS for D/L (Tables 7 and 8).

**Table 7:** Spectral data volume transferred to the Europa Clipper BDS for D/L, as a function of data cube type

Cube Type	Oversampling	Acquired Frames	Frames/Lines sent to BDS	Spectral Data Volume [Mb]		
				(uncompressed)	to BDS	for D/L
Global	20×	6000	300	645.12	430.08	430.08
Joint Scan	20×	7700	385	827.90	551.94	551.94
Regional	20×	6000	300	645.12	430.08	430.08
Local	7×	560	560	1376.26	1376.26	917.50
Dark Cal.	N/A	320	1	2.15	1.43	1.43

**Table 8:** Total data volume, including bookkeeping types, transferred to the Europa Clipper BDS for D/L, as a function of data cube type

Cube Type	Bookkeeping Type	Bookkeeping Data Volume [Mb]			Total Data Volume [Mb]	
		(uncompressed)	to BDS	for D/L	to BDS	for D/L
Global	Flag	46.08	30.72	30.72	460.80	460.80
Joint Scan	Count	354.82	236.54	236.54	788.48	788.48
Regional	Vector	1474.56	1474.56	983.04	1904.64	1413.12
Local	N/A	0.00	0.00	0.00	1376.26	917.50
Dark Cal.	Stats	1.05	1.05	1.05	2.48	2.48

When Europa Clipper is at MISE global observation altitude ranges (40,000 to 1200 km) there is adequate time to bracket each science observation with the acquisition of dark frame stacks. Closer to Europa (or other Jovian satellites) and in the MISE regional and local altitudes (below 1200 km), dark frame stacks are scheduled less frequently to maximize the available Europa observing time. Each dark frame stack is processed in the instrument DPU to produce a radiation noise remediated dark median frame, as well as a histogram of dark-corrected dark frame stack data (dark statistics). While the acquired dark frame stack is not routinely transferred to the BDS for D/L, the dark median and dark statistics are nominal D/L data products.

### On-Board Data Processing

The energetic particle flux and energy distribution at Europa’s orbital distance are significant operational considerations with respect to both the radiation budget (TID) and radiation-induced noise in the acquired science data (Man 2018). The MISE approach to radiation noise mitigation has three key components:

1. Shielding of the focal plane to reduce the energetic particle flux,
2. Operational best practices that include: (a) a short exposure time (53.47 ms) to limit the accumulation of radiation noise in any individual frame; (b) along-track oversampling of the surface using the scan system to recover the required signal; (c) frequent acquisition of dark frame stacks which sample the instrument state and variable radiation noise environment, and
3. On-instrument processing of the acquired dark frame stack and oversampled image cube data to minimize the effects of radiation noise and maximize the SNR in the frame-aggregate image cubes that are sent to the spacecraft for downlink (D/L).

Much of this data handling is done by the MISE instrument Data Processing Unit (DPU). The MISE DPU features a rank-order hardware accelerator that can sort up to 32 samples and calculate the mean and/or median from a selected continuous subset of the rank-ordered elements (high- and/or low- tail exclusion). The DPU data processing capability design target is an assumed 15% per frame radiation noise event probability above the signal-independent instrument noise envelope, with reference to a radiation noise DN distribution traceable to beam line testing and integrated instrument radiation transport modeling.

#### Dark Frame Sampling Within the MISE Reference Encounter

An illustration of the MISE 10-cube reference Europa encounter scenario is shown in Fig. 26. Every stack of dark frames [D] is processed to generate a set of D/L products consisting of (1) a single radiation noise mitigated aggregate dark frame that is used in the MISE ground radiometric calibration; and (2) a dark-corrected dark frame stack histogram that supports the evaluation of instrument performance and the characterization of the radiation noise environment. Selected dark frame stack processing results also inform the data processing. Every global-scale [G], joint scan [J], and regional-scale [R] oversampled observation is processed to generate D/L products consisting of: (1) a radiation noise remediated and spatially regularized aggregate spectral image cube; and (2) an optional bookkeeping image cube that records additional information about the as-acquired scene data and the DPU data processing.

#### Dark Stack Processing

With the MISE dark-stack processing method, the locations and amounts of radiation noise are based on onboard comparison with large sampling of “dark” observations that have been collected in very close temporal proximity to the science or calibration cube, and thus no pre-assumed statistic look-up tables are needed, and the performance of each detector element is checked for each observation. The data processing objectives for each stack of dark frames (nominally 320 frames) acquired before and after each observation cube, or as close in time as possible (while in a comparable radiation environment), are: (1) generate a single aggregate dark frame; (2) gather instrument and radiation noise statistics; and (3) calculate an effective radiation noise hit rate table for the observed radiation noise distribution.

**Fig. 27:**
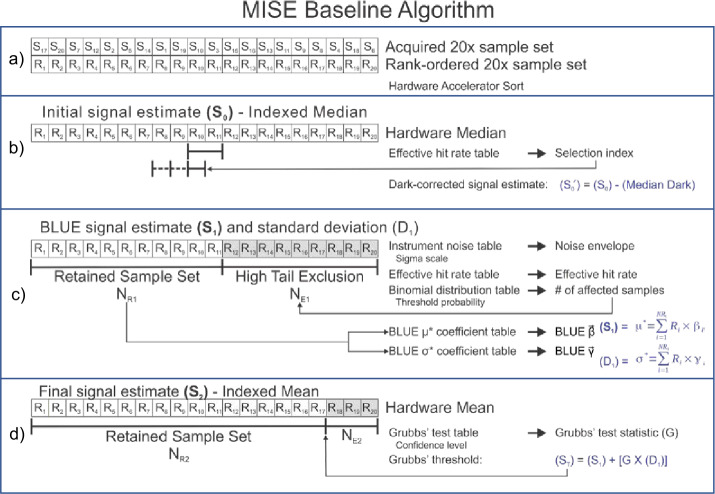
The sequence of processing steps within the MISE baseline frame aggregation and radiation noise mitigation. Starting with the oversampled observation (a) and related dark calibration observations, the results are dark-corrected (b) and statistics on the noise are generated (c). The final cube (d) would have radiation noise removed

To remove radiation noise, the single aggregate dark frame is meant to represent a median dark value across the 320 samples in the input frame stack, but since the hardware accelerator is limited to 32 samples, a “cascade median” is computed from the median of 10 sets of 32 samples, and then the median of those 10 results (Fig. 27a, b). This aggregate dark frame is subtracted from each frame in the dark stack, yielding the dark-corrected dark stack data. A histogram of the dark-corrected dark stack data distribution is then used to determine the instrument and radiation noise statistics. An effective hit rate table—that is, the fraction of pixels in the frame stack that experienced a radiation noise event at a given DN level or above—is calculated as the reverse cumulative distribution of the isolated radiation noise distribution. The effective hit rate table and aggregate dark frame are used to process the image cube.

#### Image Cube Processing

For each oversampled observation (nominally 6000 frames; 300 lines at 20× oversampling) data processing is used to (1) generate an aggregate regularly sampled image cube where each subset of spatially oversampled frames [x, $\lambda $, t_a_:t_b_] have been combined to produce an aggregate frame [x, $\lambda $, t_k_] that maximizes the SNR and minimizes the influence of radiation noise on the aggregate result. The frame-to-frame variability of the radiation noise, the governing distributions for the radiation noise event probability and amplitude, and relatively large (20×) nominal oversampling factor allow each aggregate element [x_i_, $\lambda _{\mathrm{j}}$, t_k_] to be derived from a subset of continuous samples in the acquired data cube [x_i_, $\lambda _{\mathrm{j}}$, t_a_:t_b_], traceable to the corresponding individual detector element [x_i_, $\lambda _{\mathrm{j}}$]. The evaluation of each 1D sample set is supported by a collection of statistical and instrument characterization look-up tables calculated in advance that are stored in the instrument memory, plus the associated effective hit rate table and aggregate dark frame derived on board (Fig. 27c). The extensive use of look-up tables expedites the data processing by minimizing the number of required DPU software calculations.

In addition, bookkeeping information may be generated within the data processing to record characteristics of the acquired oversampled data, the algorithm processing, and/or the aggregate result.

The sample set evaluation progresses through a three- part series of increasingly rigorous estimates of the radiation noise remediated aggregate result. Uncertainty in the result can be traded against processing time by truncating the calculation at an intermediate step. The initial signal estimate (S_0_) is the median of the sample set calculated using only the hardware accelerator. This initial signal level is dark-corrected using the aggregate dark frame and the corrected value along with a specified sigma level indexes an instrument radiometric model noise table to establish the instrument noise envelope. The width of the noise envelope in turn indexes the effective radiation noise hit rate table to establish the effective radiation noise event probability for the sample set. This sample-set-specific event probability along with a specified tolerance then indexes a binomial distribution table which returns the number of samples that are expected to be unaffected by a radiation noise event in excess of the noise envelope to the specified tolerance. This series of table look-ups allows for the calculation of an order-statistics model signal (S_1_) as the best linear unbiased estimate (BLUE) of the location parameter ($\mu $) for a Type II censored sample set (Gupta 1952). The normal order statistics BLUE coefficients for the set of possible censored sample set configurations are also stored in look-up tables. With the model estimates of the radiation-noise-free sample set mean and standard deviation, an outlier threshold is calculated by looking up the one-sided Grubbs’ test statistic (Grubbs 1969) in a table indexed by sample size and a specified confidence level. Returning to the rank-order hardware accelerator, the mean of all samples less than the outlier threshold is returned as the final result (S_2_). Simulation of MISE acquired dark frame stacks, oversampled scene data, and the corresponding resultant data products has demonstrated that the aggregate science data product requirements (SNR, data volume) are met with the available processing resources (energy, time).

#### Data Processing Bookkeeping

The variation in the number of acquired data samples participating in the calculation of a given aggregate element means the SNR will vary element-to-element in the aggregate cube, and the variation in the index position of the participating data samples means the effective along-track spatial sampling function will also vary. This interesting characteristic of the MISE aggregate data motivates a set of bookkeeping products which record additional information about each aggregate element at different levels of detail. The bookkeeping options include: (1) flag cube (boolean)—records if the number of samples that contributed to each aggregate element exceeds a given threshold (typically corresponding to the SNR requirement); (2) count cube (integer)—records the number of samples that contributed to each aggregate element (per-element SNR); (3) vector map (long integer)—documents which samples were excluded (per-element along-track sampling function).

### Engineering Data

Depending on the mode that MISE is operating in, engineering data detailing instrument health and status will be streamed at a high or low rate. The high rate is 1 Hz, and the low rate is once per minute. A single health and status packet is 2400 bits in size and a single engineering data packet is approximately 2400 bits in size. This makes an entire packet size for engineering data of 4800 bits.

## Conclusion

The scientific goal of the Europa Clipper mission is to determine whether there are places below Europa’s surface that could support life. Europa shows strong evidence of the presence of a liquid water ocean beneath an icy shell, and so is considered a promising currently habitable environment in the Solar System. The ocean might have all the requirements needed for life as we know it: water, organics, energy and stability. The MISE instrument—a Dyson infrared mapping spectrometer—will play a major role in investigating Europa’s habitability by using infrared spectroscopy to map the distribution of salts, organics, radiolytic products, surface ice structure, and hot spots on Europa where recent or active resurfacing may be taking place. MISE will also make close-range observations of Ganymede and Callisto during flybys, and longer-range observations of Jupiter and Io. Analysis of the spectral observations by MISE will yield the most comprehensive composition maps of Europa ever obtained, significantly advancing our understanding of this unique moon.
